# Supplementary material for: Ezh2 Loss-of-Function Alters Zebrafish Cerebellum Development
Source: Int J Mol Sci. 2025 Oct 7;26(19):9736. doi: 10.3390/ijms26199736 (PMC12524611; doi:10.3390/ijms26199736)
Supplement: Supplementary file 1 [file ijms-26-09736-s001.zip › ijms-3873631-supplementary.pdf]

Ezh2 loss-of-function alters zebrafish cerebellum development

Mariette Hanot, Pamela Völkel, Xuefen Le Bourhis, Chann Lagadec and Pierre-Olivier Angrand

Supplementary information

Supplementary Figure S1: Whole-mount RNA *in situ* hybridization for *olig2* at 2 dpf
Supplementary Figure S2: Whole-mount RNA *in situ* hybridization for *olig2* at 3 dpf
Supplementary Figure S3: Whole-mount RNA *in situ* hybridization for *olig2* at 5 dpf
Supplementary Figure S4: Whole-mount RNA *in situ* hybridization for *mag* at 5 dpf
Supplementary Figure S5: Whole-mount RNA *in situ* hybridization for *mpz* at 5 dpf
Supplementary Figure S6: Whole-mount RNA *in situ* hybridization for *pcna* at 2 dpf
Supplementary Figure S7: Whole-mount RNA *in situ* hybridization for mag at 3 dpf
Supplementary Figure S8: Whole-mount RNA *in situ* hybridization for *pcna* at 5 dpf
Supplementary Figure S9: Whole-mount RNA *in situ* hybridization for *ccna2* at 5 dpf
Supplementary Figure S10: Whole-mount RNA *in situ* hybridization for *atoh1a* at 2 dpf
Supplementary Figure S11: Whole-mount RNA *in situ* hybridization for *atoh1a* at 5 dpf
Supplementary Figure S12: Whole-mount RNA *in situ* hybridization for *atoh1c* at 2 dpf
Supplementary Figure S13: Whole-mount RNA *in situ* hybridization for *atoh1c* at 5 dpf
Supplementary Figure S14: Whole-mount RNA *in situ* hybridization for *ptf1a* at 2 dpf
Supplementary Figure S15: Whole-mount RNA *in situ* hybridization for *neurod1* at 3 dpf
Supplementary Figure S16: Whole-mount RNA *in situ* hybridization for *neurod1* at 5 dpf
Supplementary Figure S17: Whole-mount RNA *in situ* hybridization for *vglut1* at 3 dpf
Supplementary Figure S18: Whole-mount RNA *in situ* hybridization for *vglut1* at 5 dpf
Supplementary Figure S19: Whole-mount RNA *in situ* hybridization for *pvalb7* at 3 dpf
Supplementary Figure S20: Whole-mount RNA *in situ* hybridization for *pvalb7* at 5 dpf
Supplementary Figure S21: Whole-mount RNA *in situ* hybridization for *gad1b* at 5 dpf
Supplementary Figure S22: Whole-mount RNA *in situ* hybridization for *slc18a2* at 5 dpf
Supplementary Figure S23: Whole-mount RNA *in situ* hybridization for *tph2* at 5 dpf
Supplementary Figure S24: Whole-mount RNA *in situ* hybridization for *th* at 5 dpf
Supplementary Figure S25: Whole-mount RNA *in situ* hybridization for *ptf1a* at 5 dpf
Supplementary Figure S26: Whole-mount RNA *in situ* hybridization for *pax2a* at 5 dpf
Supplementary Figure S27: Locomotor activity at 5 dpf
Supplementary Figure S28: Phenotype of *ezh2*$^{+/-}$ heterozygotes

olig2 at 2 dpf

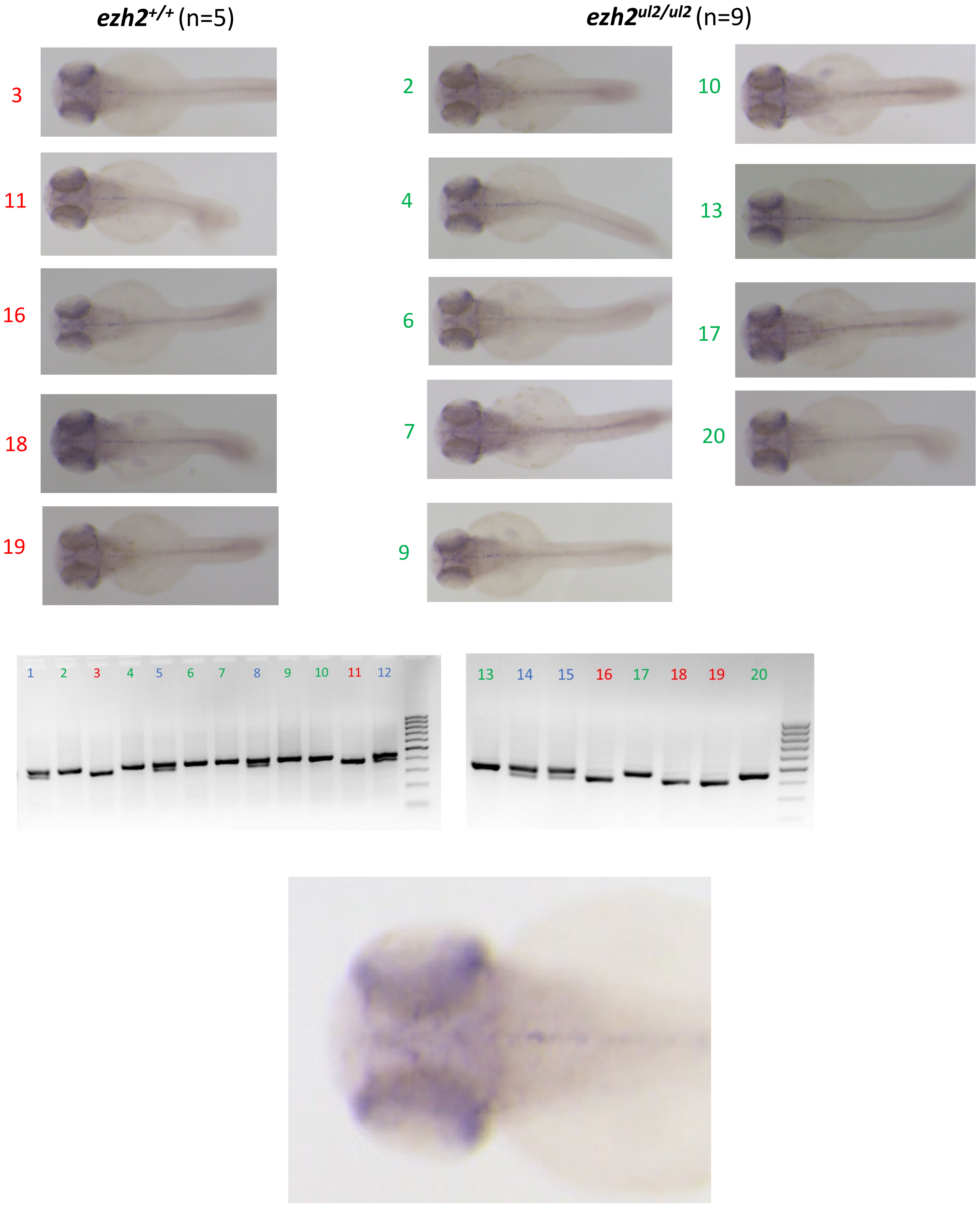

Supplementary Figure S1: Whole-mount RNA *in situ* hybridization for *olig2* at 2 dpf

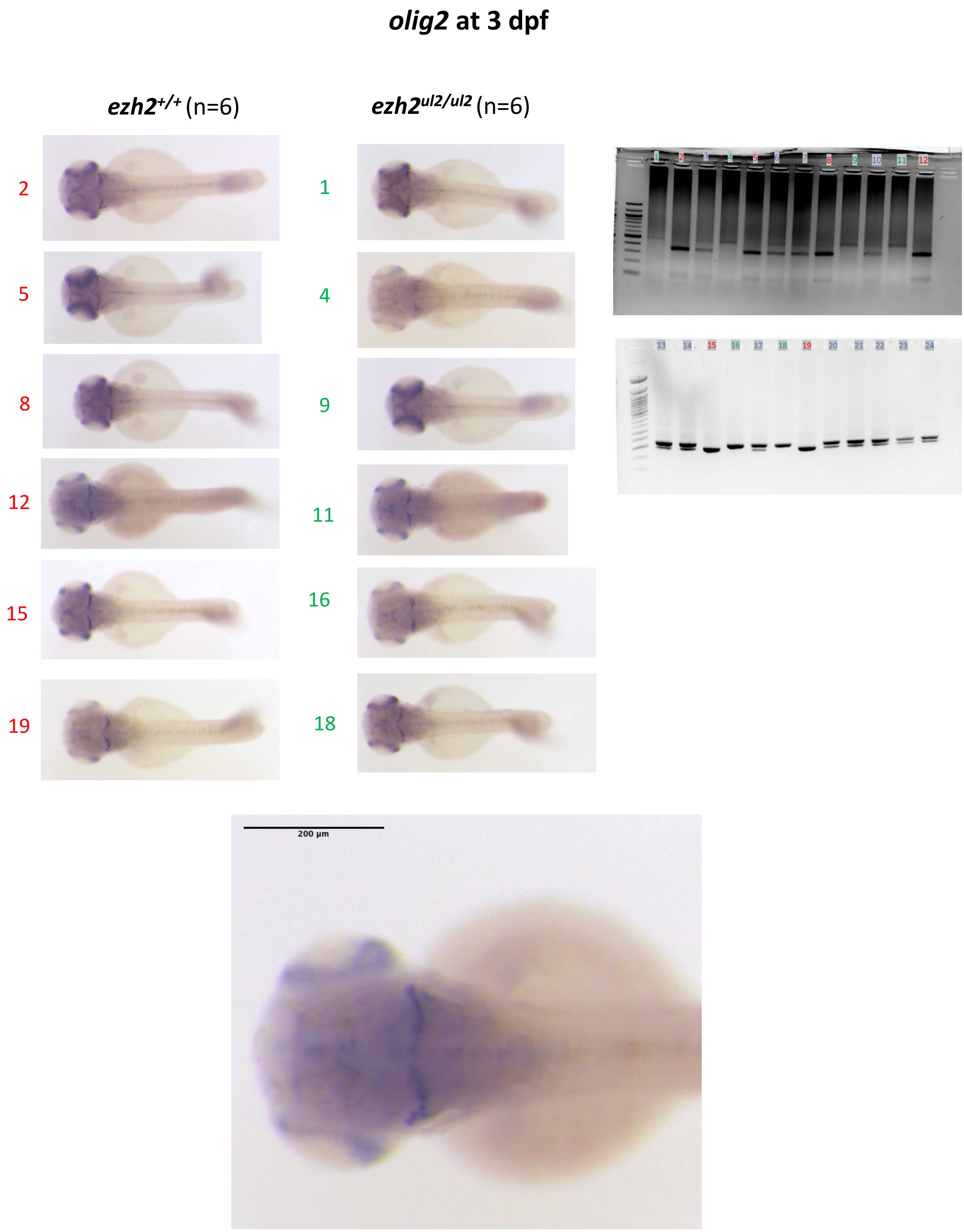

Supplementary Figure S2: Whole-mount RNA *in situ* hybridization for *olig2* at 3 dpf

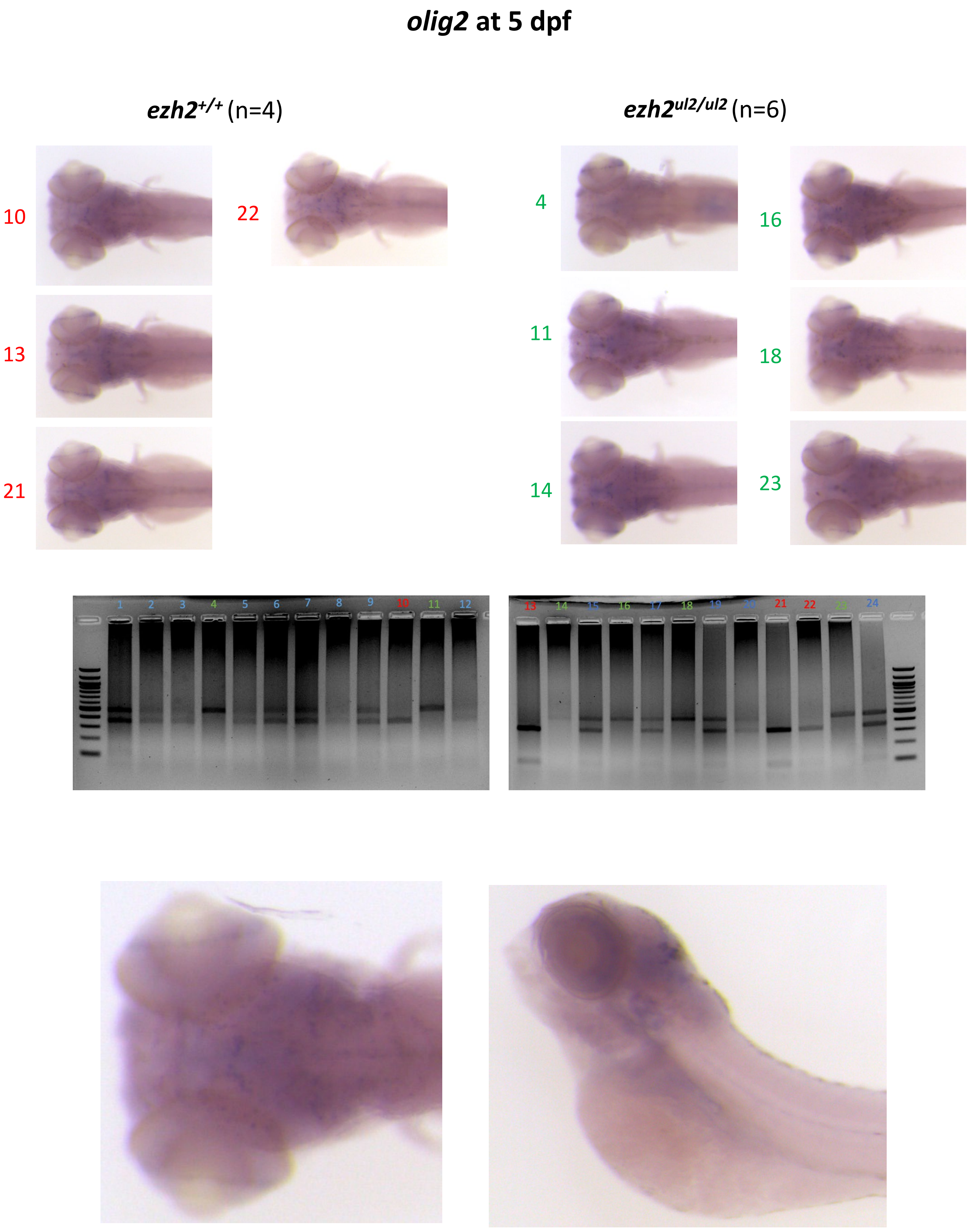

Supplementary Figure S3: Whole-mount RNA *in situ* hybridization for *olig2* at 5 dpf

mag at 5 dpf

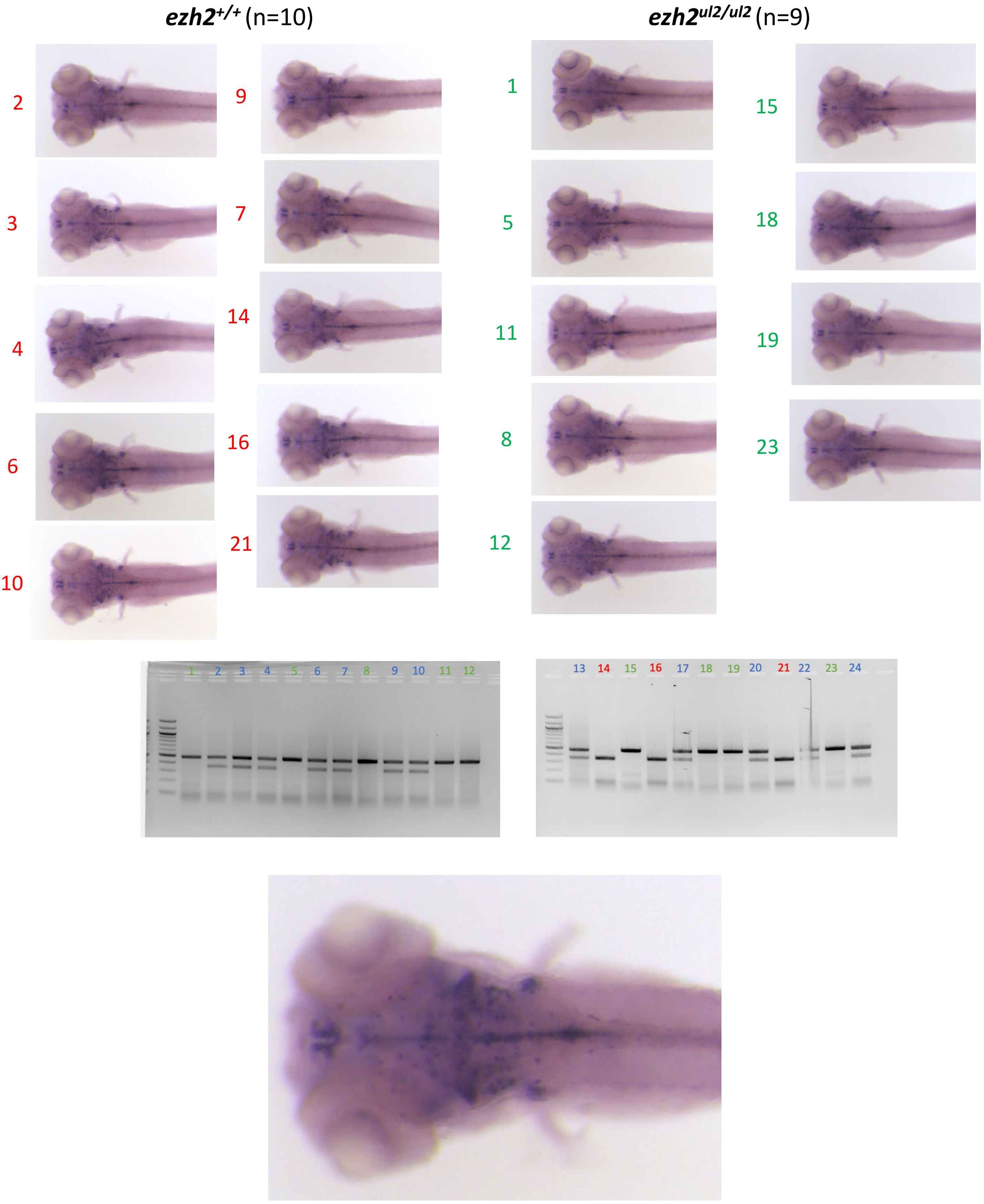

Supplementary Figure S4: Whole-mount RNA *in situ* hybridization for *mag* at 5 dpf

mpz at 5 dpf

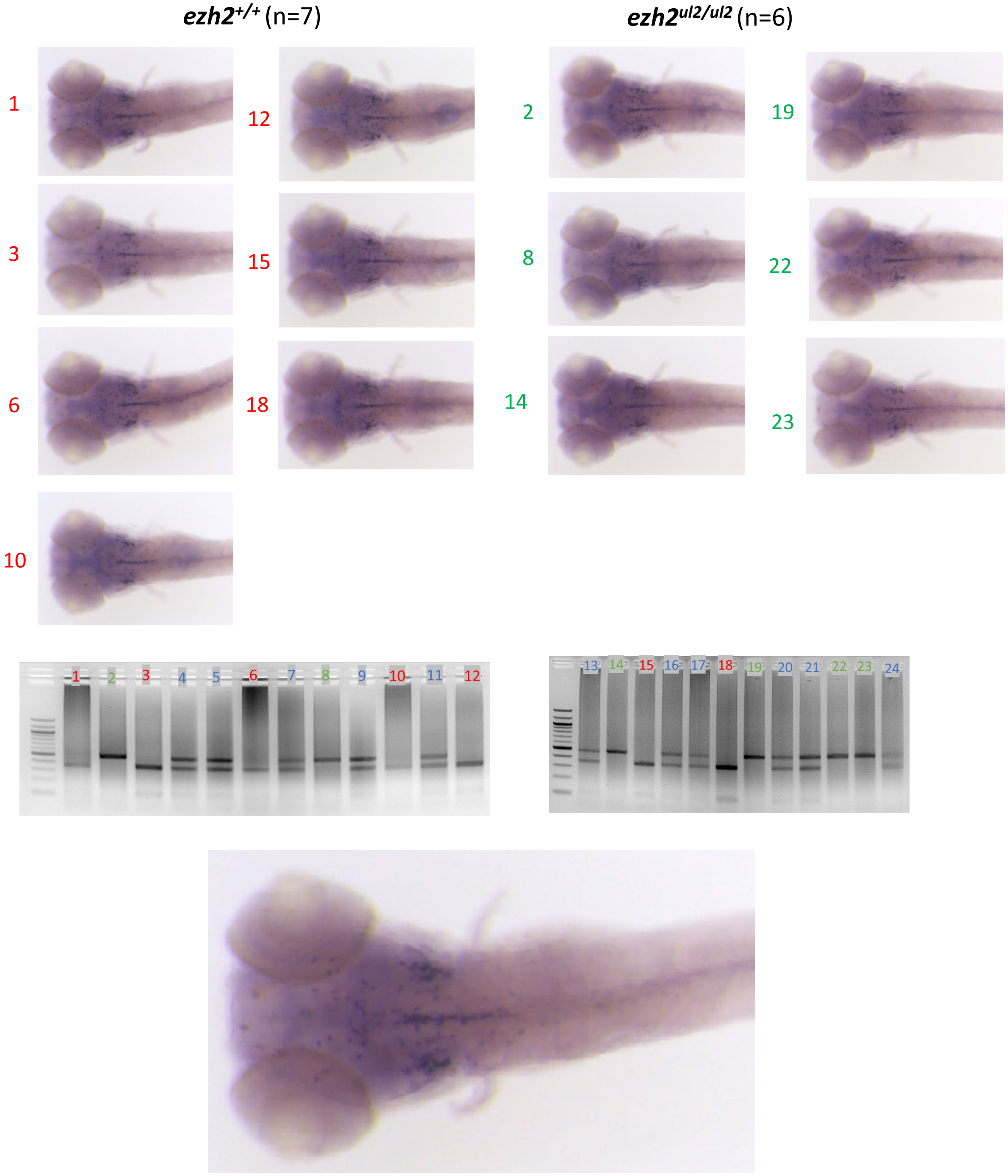

Supplementary Figure S5: Whole-mount RNA *in situ* hybridization for *mpz* at 5 dpf

pcna at 2 dpf

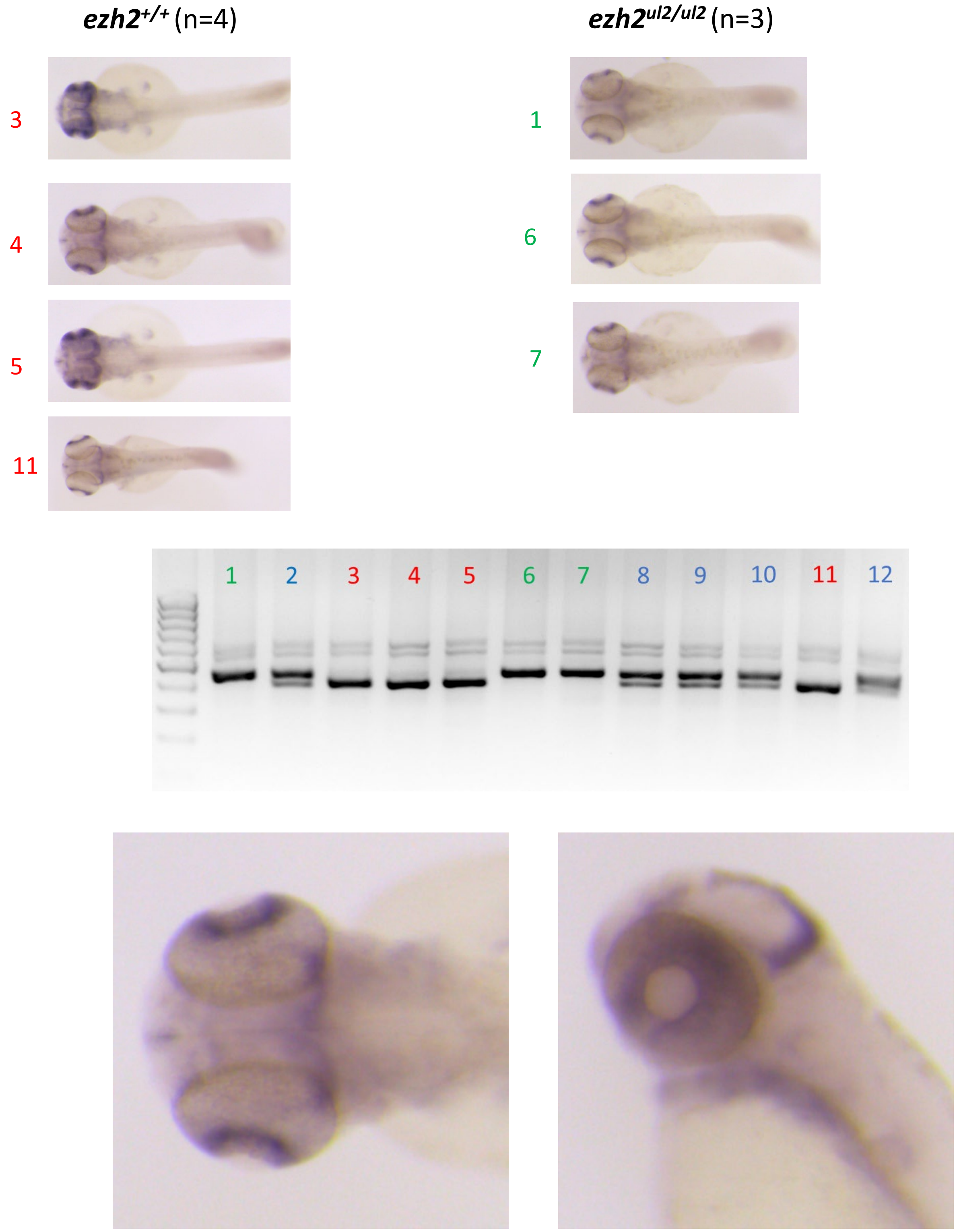

Supplementary Figure S6: Whole-mount RNA *in situ* hybridization for *pcna* at 2 dpf

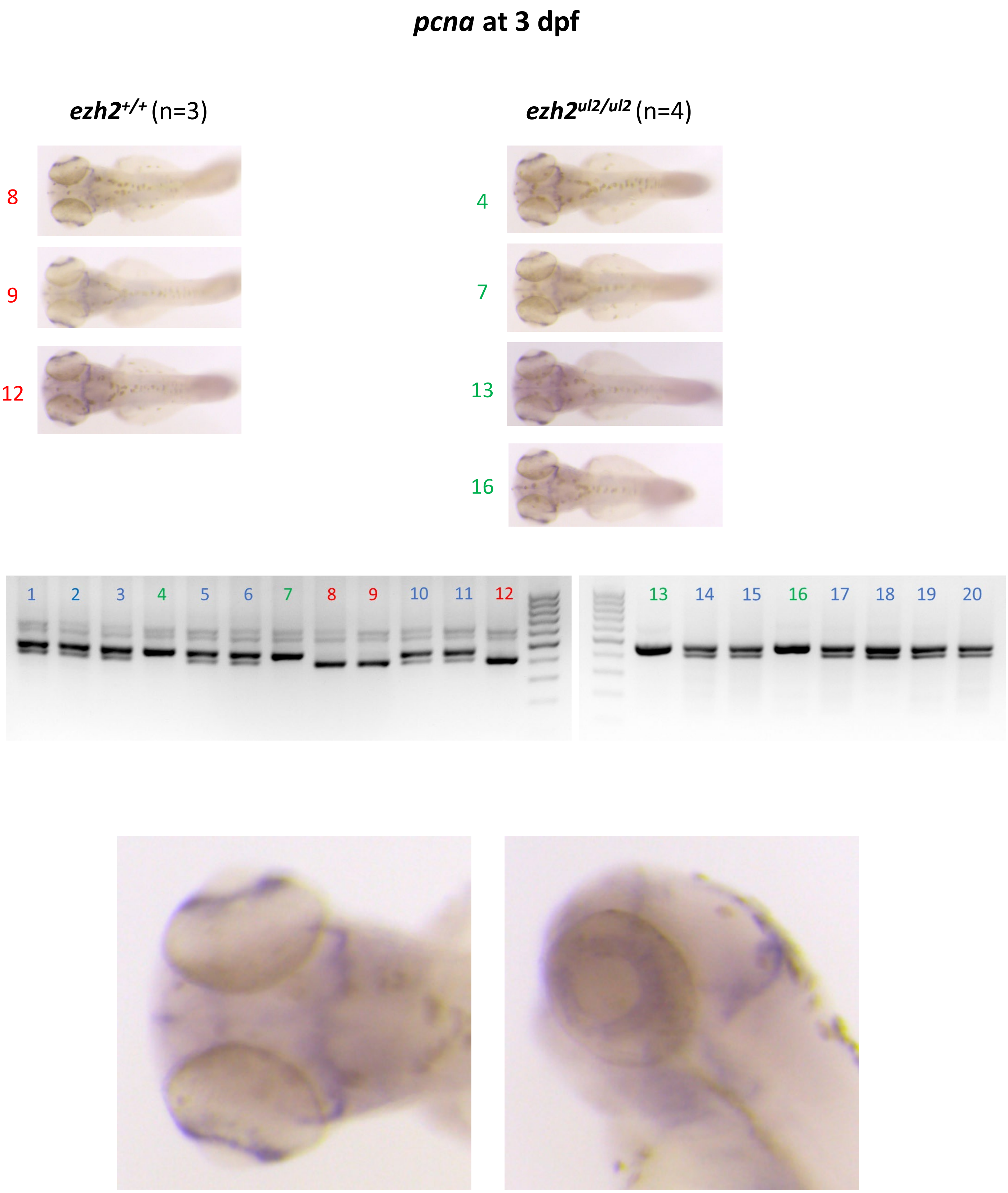

Supplementary Figure S7: Whole-mount RNA *in situ* hybridization for *mag* at 3 dpf

pcna at 5 dpf

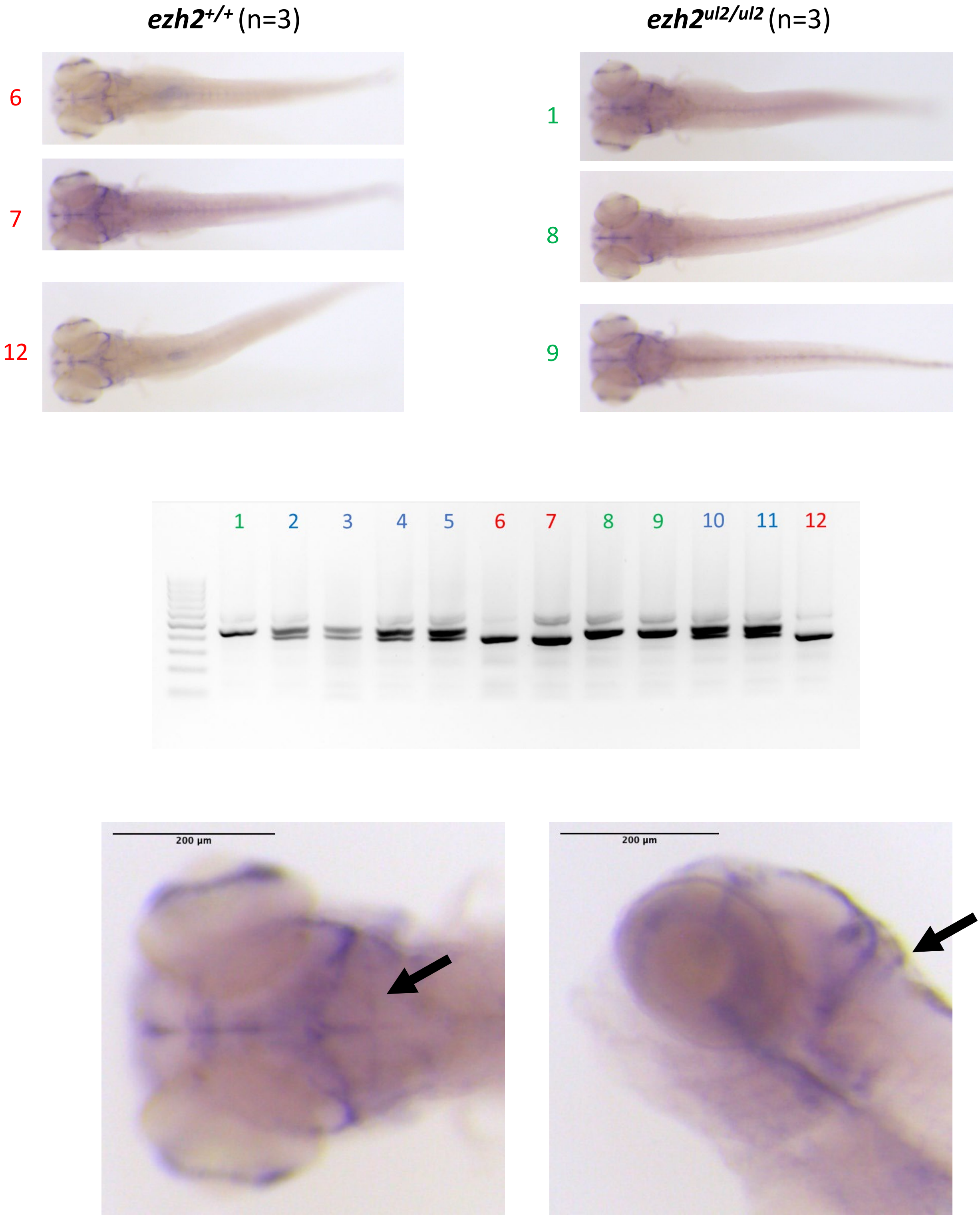

Supplementary Figure S8: Whole-mount RNA *in situ* hybridization for *pcna* at 5 dpf

ccna2 at 5 dpf

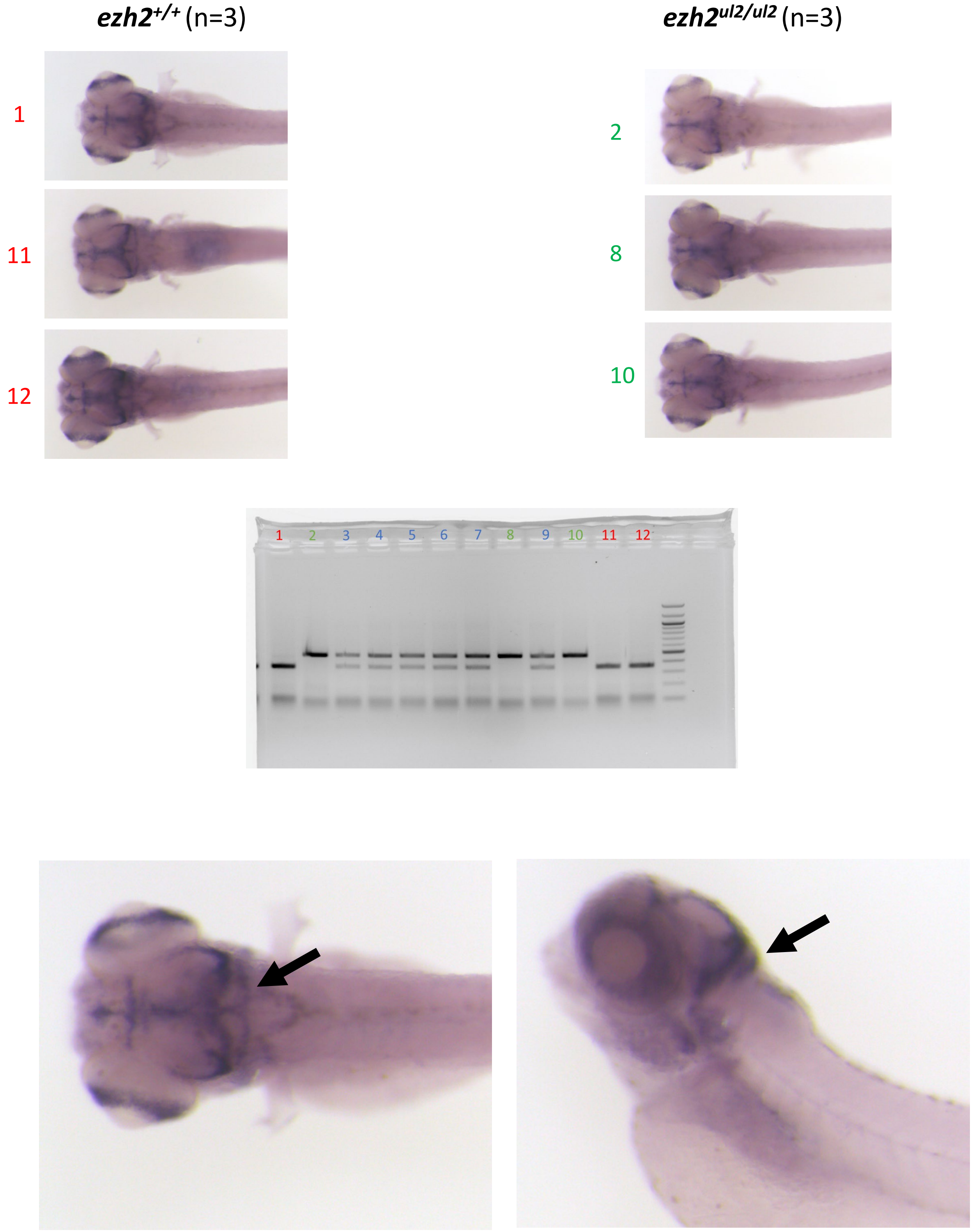

Supplementary Figure S9: Whole-mount RNA *in situ* hybridization for *ccna2* at 5 dpf

atoh1a at 2 dpf

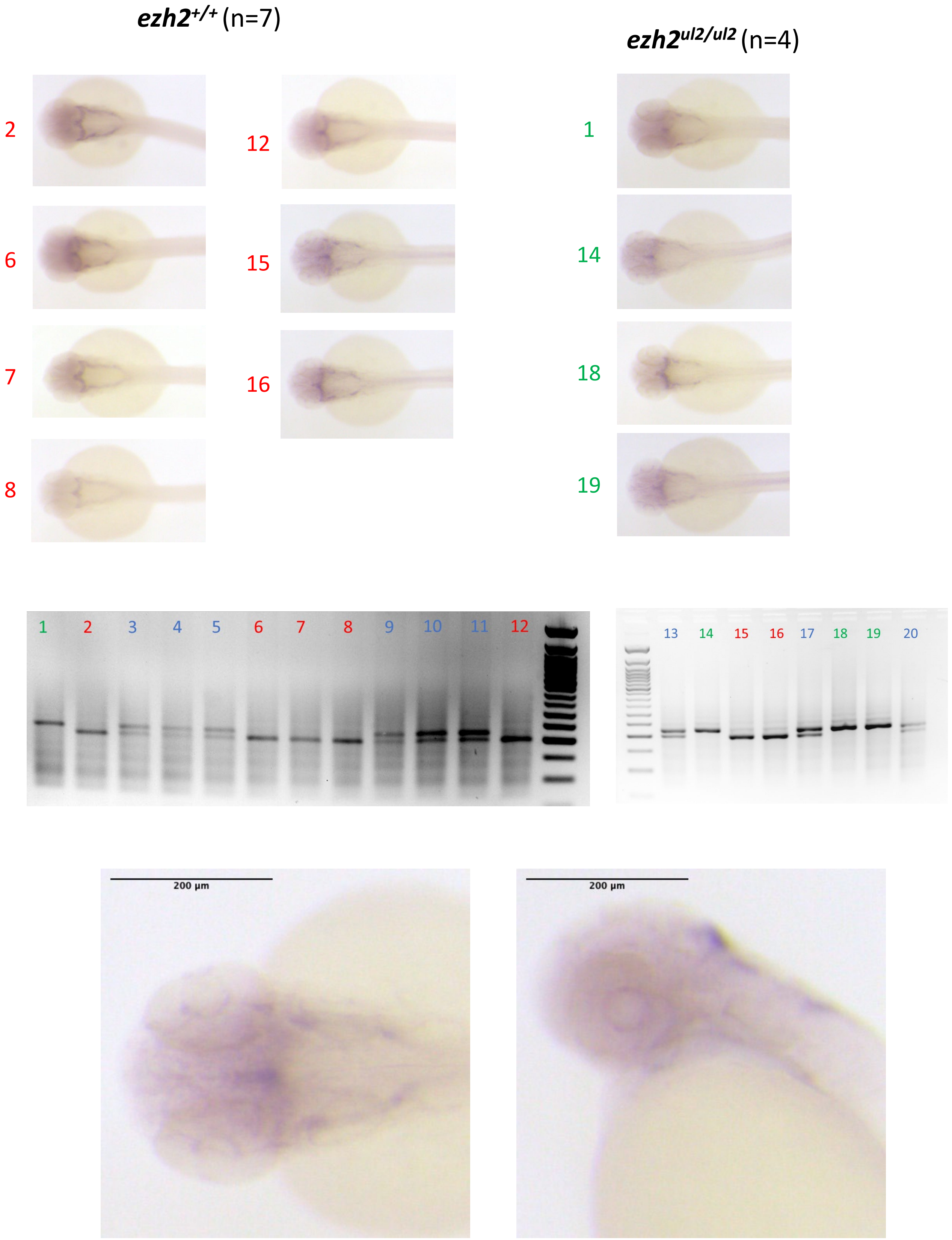

Supplementary Figure S10: Whole-mount RNA *in situ* hybridization for *atoh1a* at 2 dpf

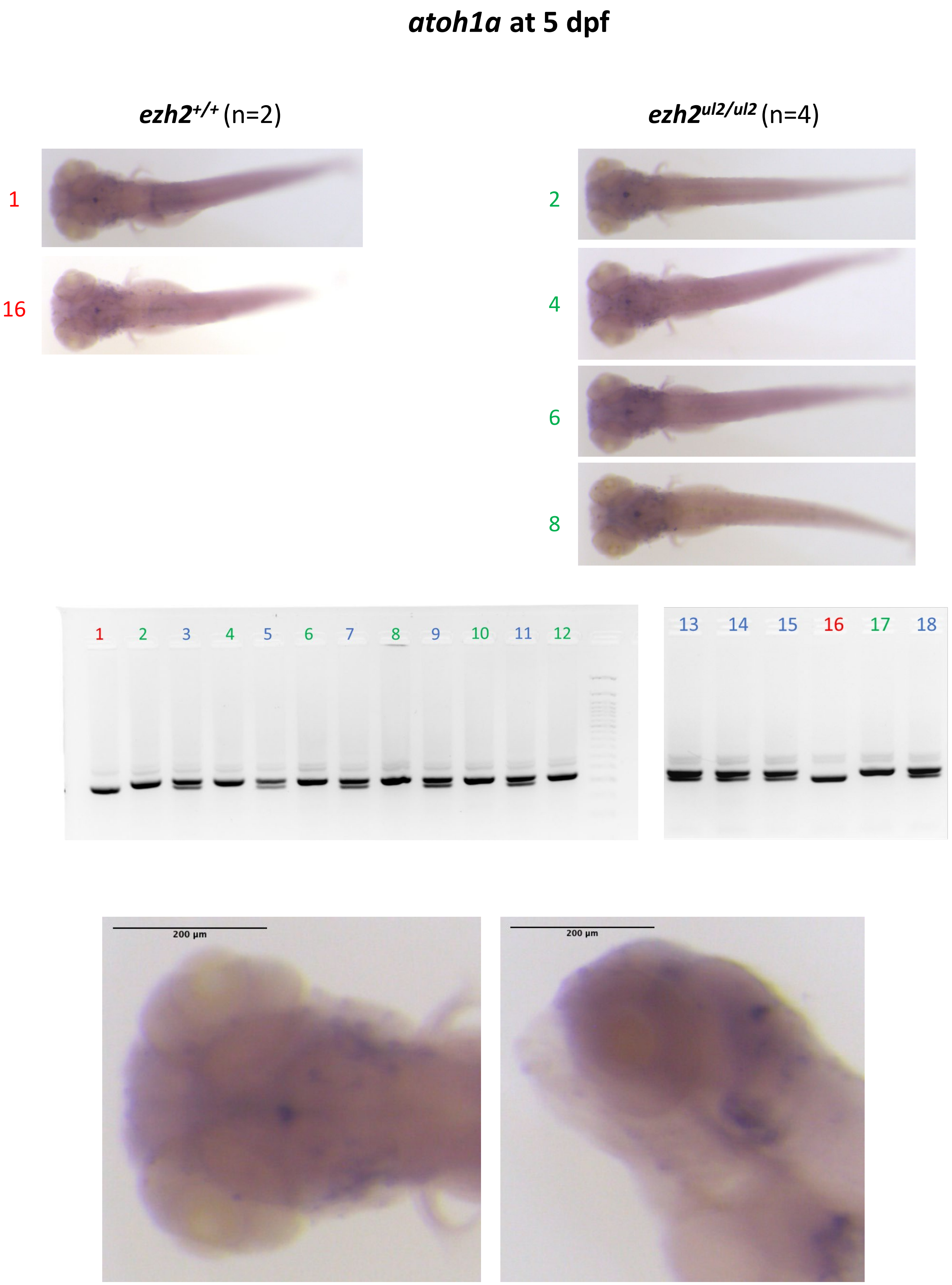

Supplementary Figure S11: Whole-mount RNA *in situ* hybridization for *atoh1a* at 5 dpf

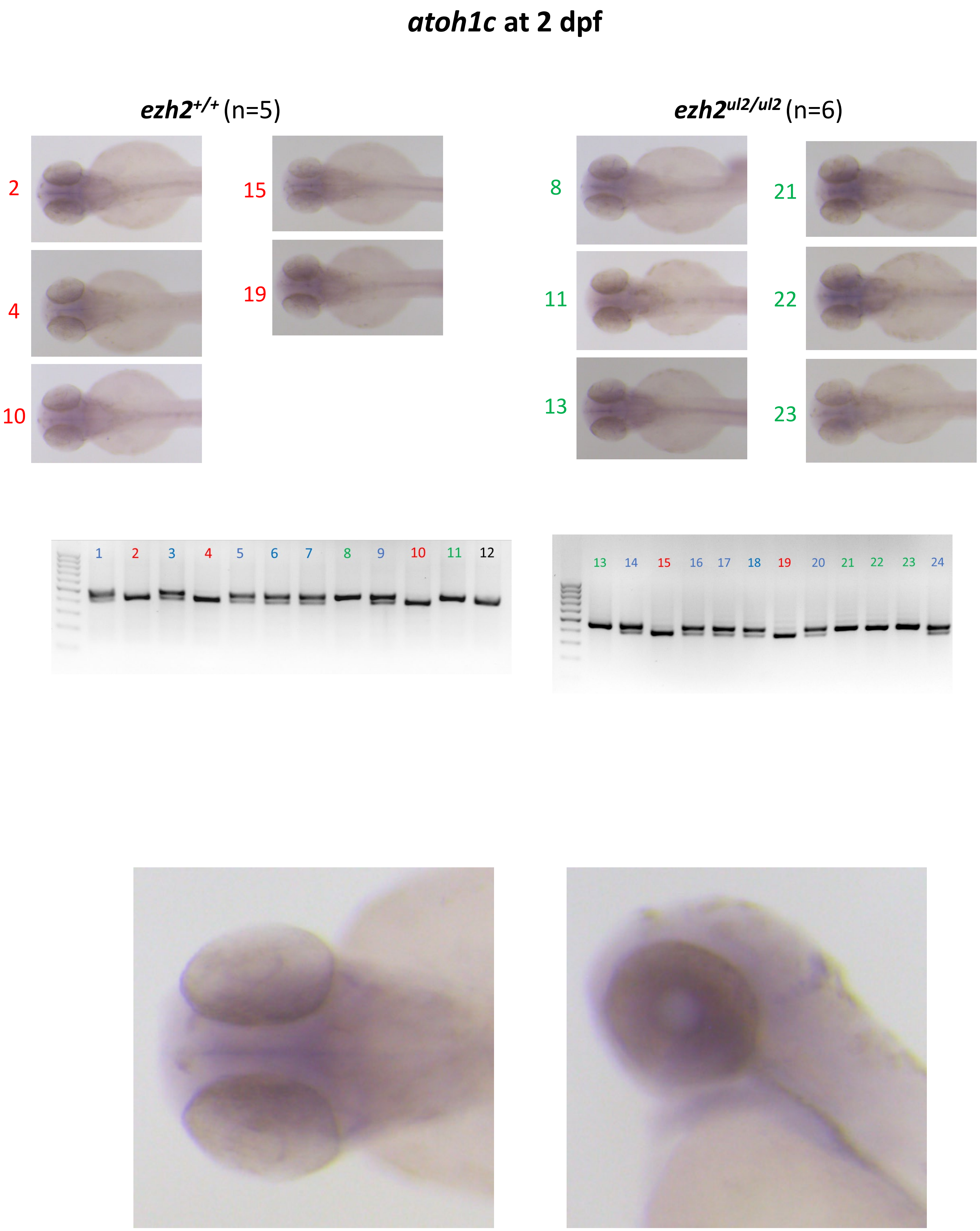

Supplementary Figure S12: Whole-mount RNA *in situ* hybridization for *atoh1c* at 2 dpf

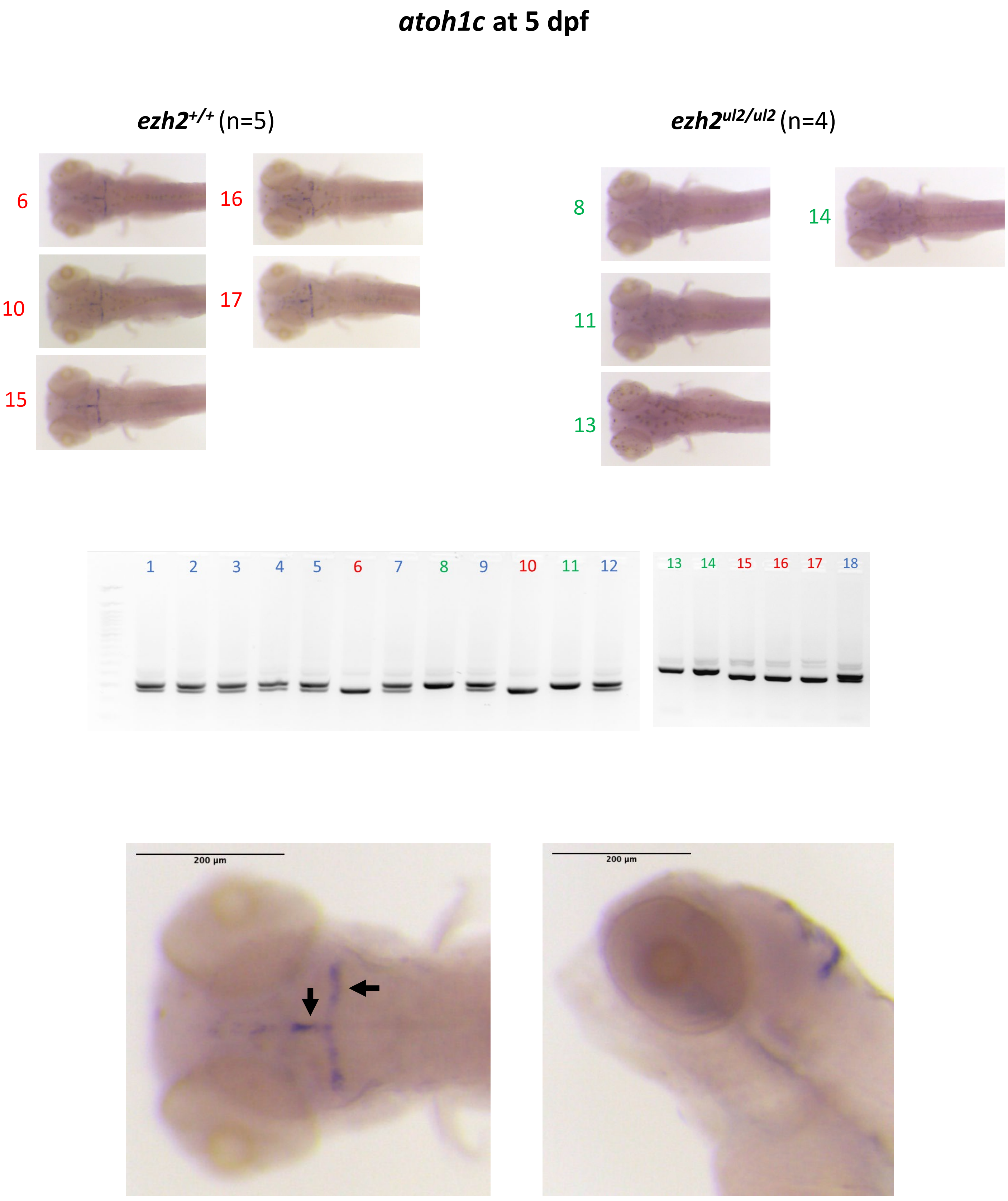

Supplementary Figure S13: Whole-mount RNA *in situ* hybridization for *atoh1c* at 5 dpf

ptf1a at 2 dpf

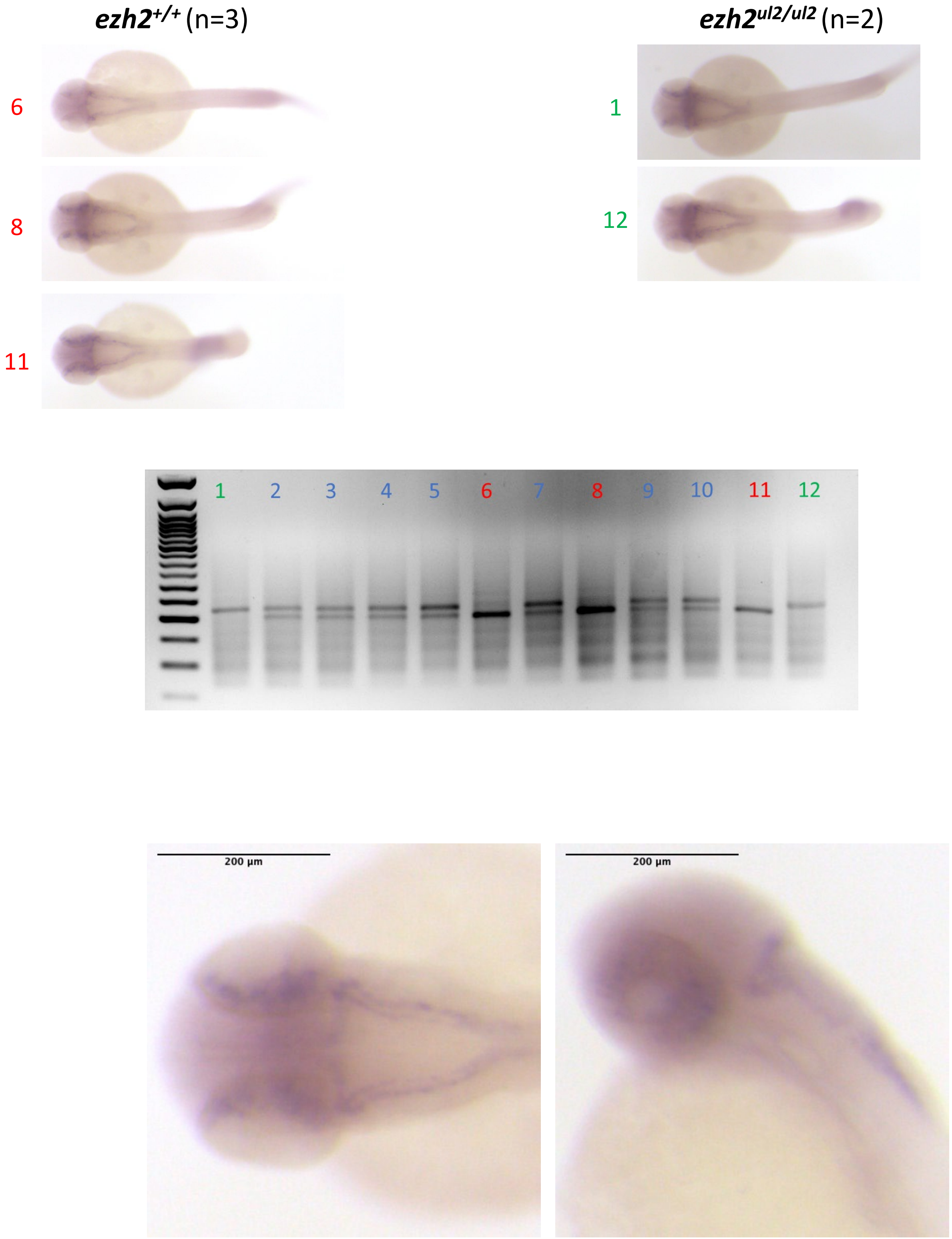

Supplementary Figure S14: Whole-mount RNA *in situ* hybridization for *ptf1a* at 2 dpf

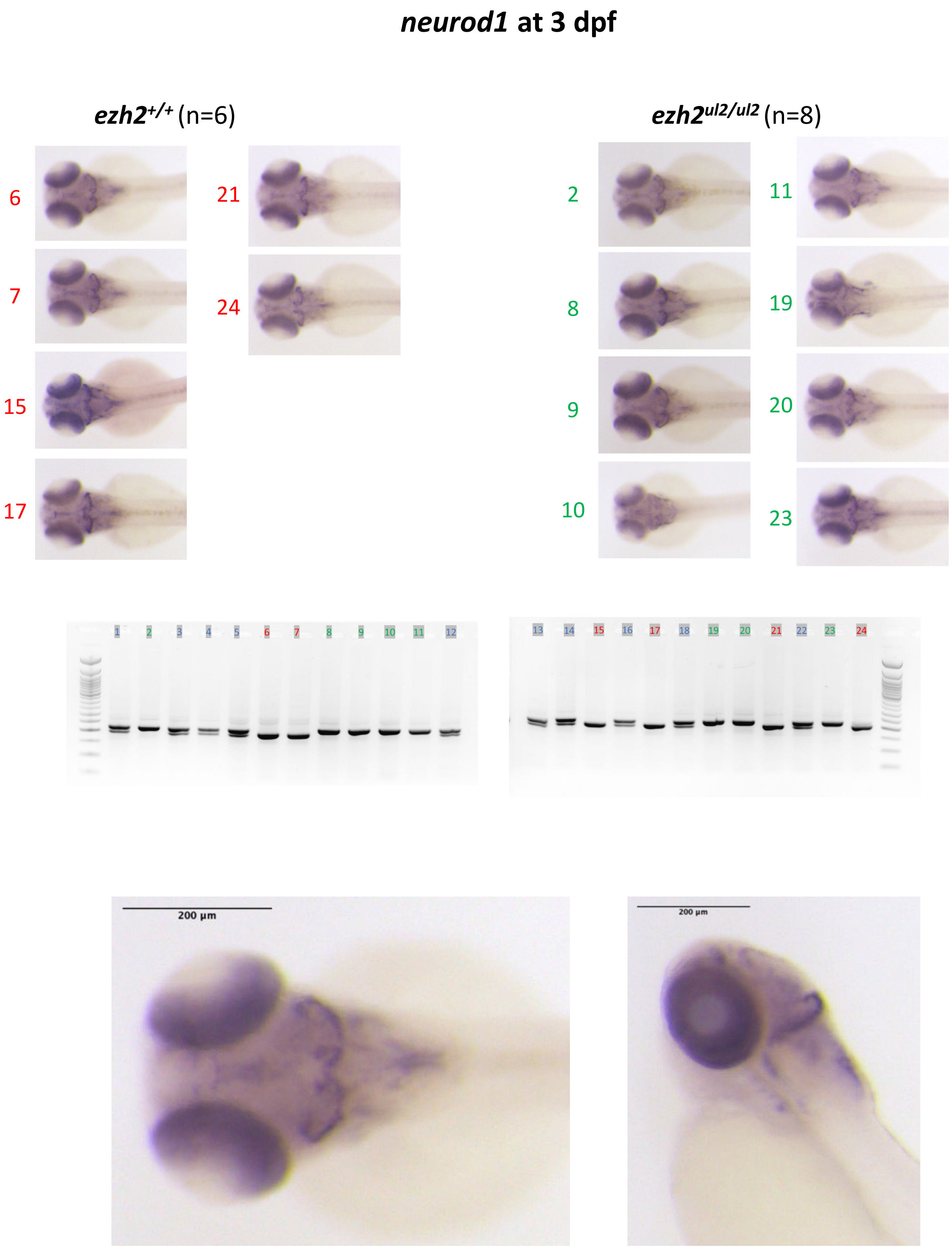

Supplementary Figure S15: Whole-mount RNA *in situ* hybridization for *neurod1* at 3 dpf

neurod1 at 5 dpf

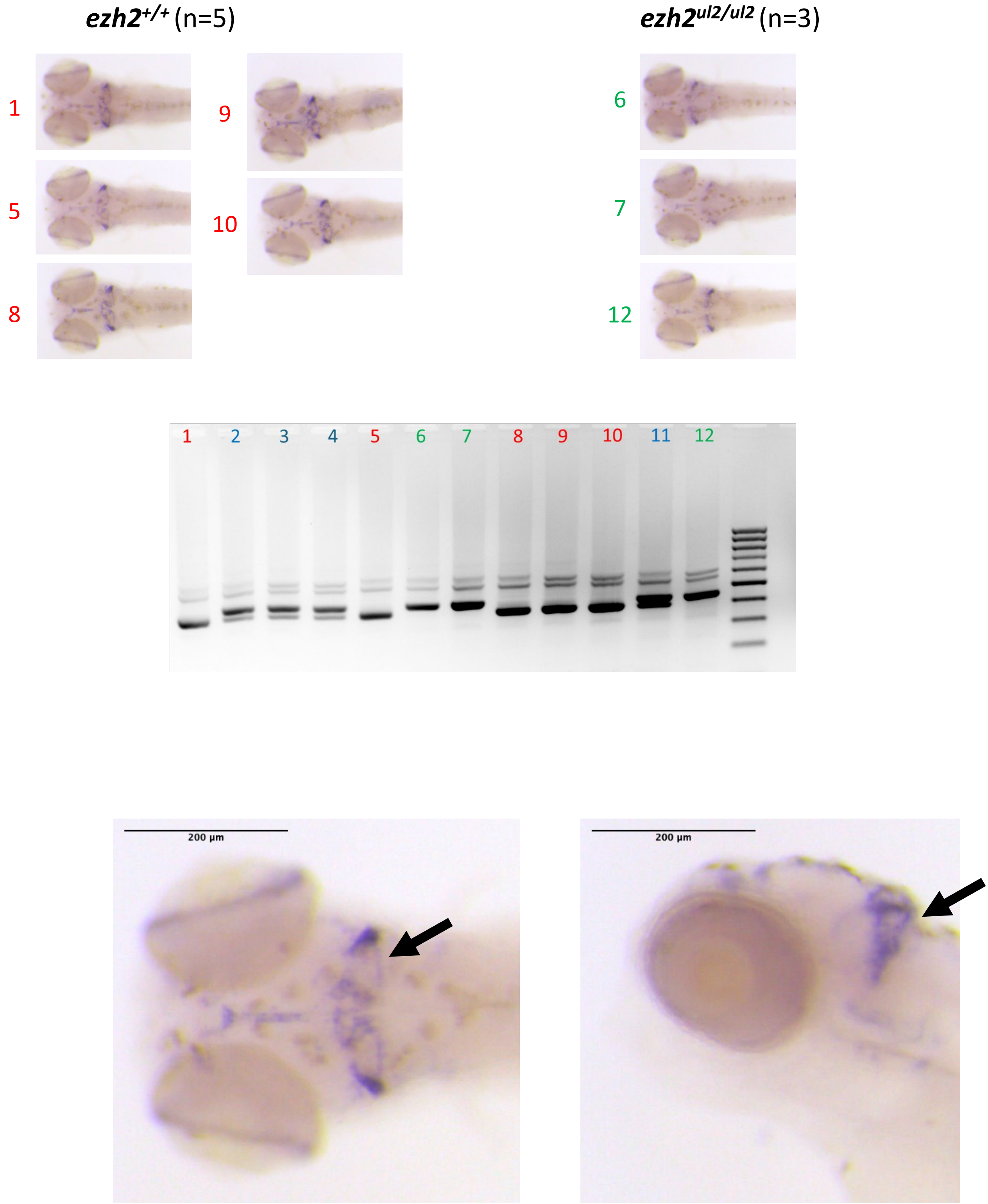

Supplementary Figure S16: Whole-mount RNA *in situ* hybridization for *neurod1* at 5 dpf

vglut1 at 3 dpf

ezh2$^{+/+}$ (n=5) ***ezh2***$^{ul2/ul2}$ (n=4)

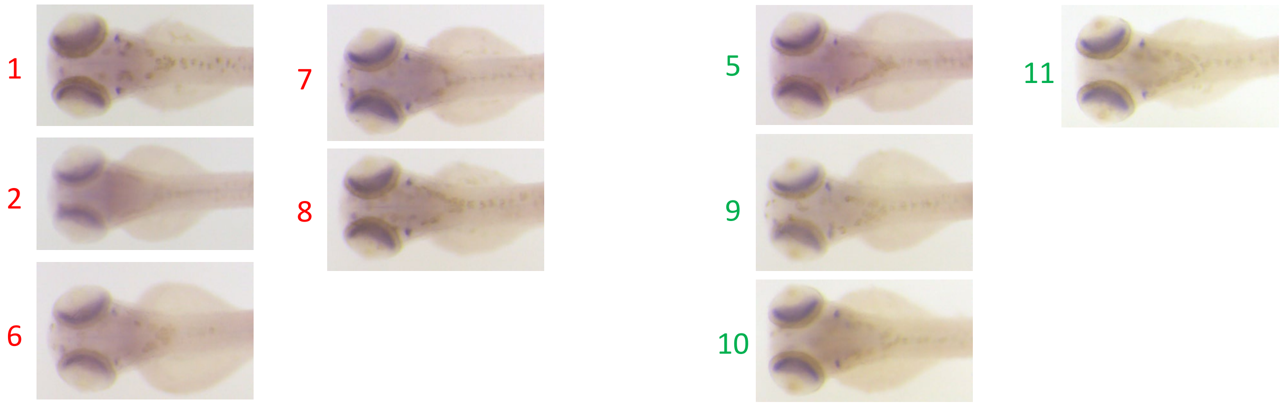

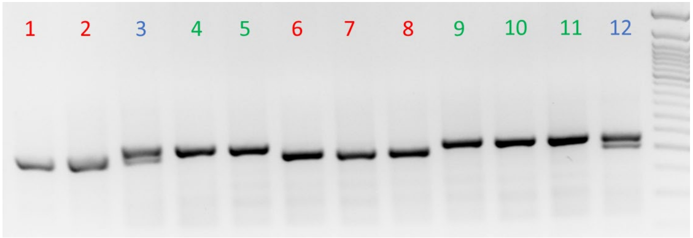

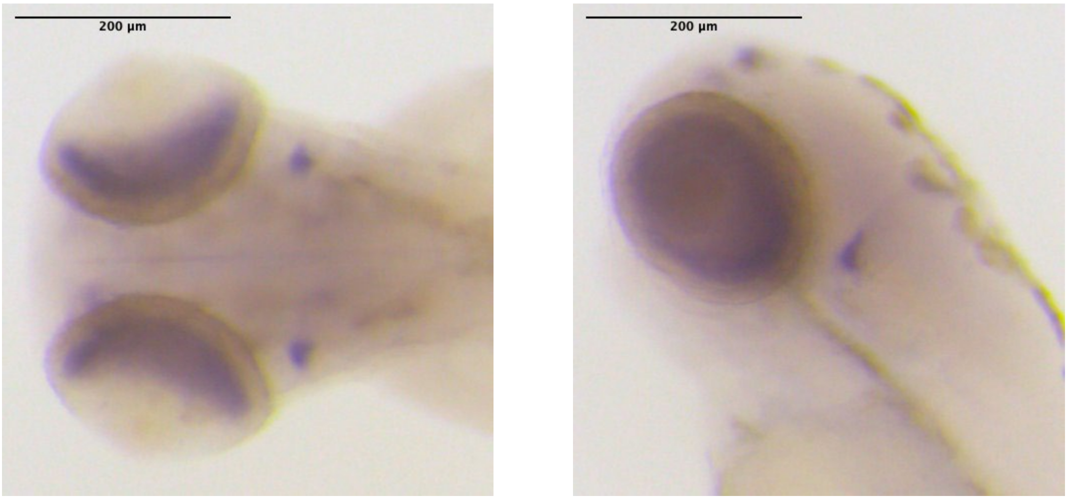

Supplementary Figure S17: Whole-mount RNA *in situ* hybridization for *vglut1* at 3 dpf

vglut1 at 5 dpf

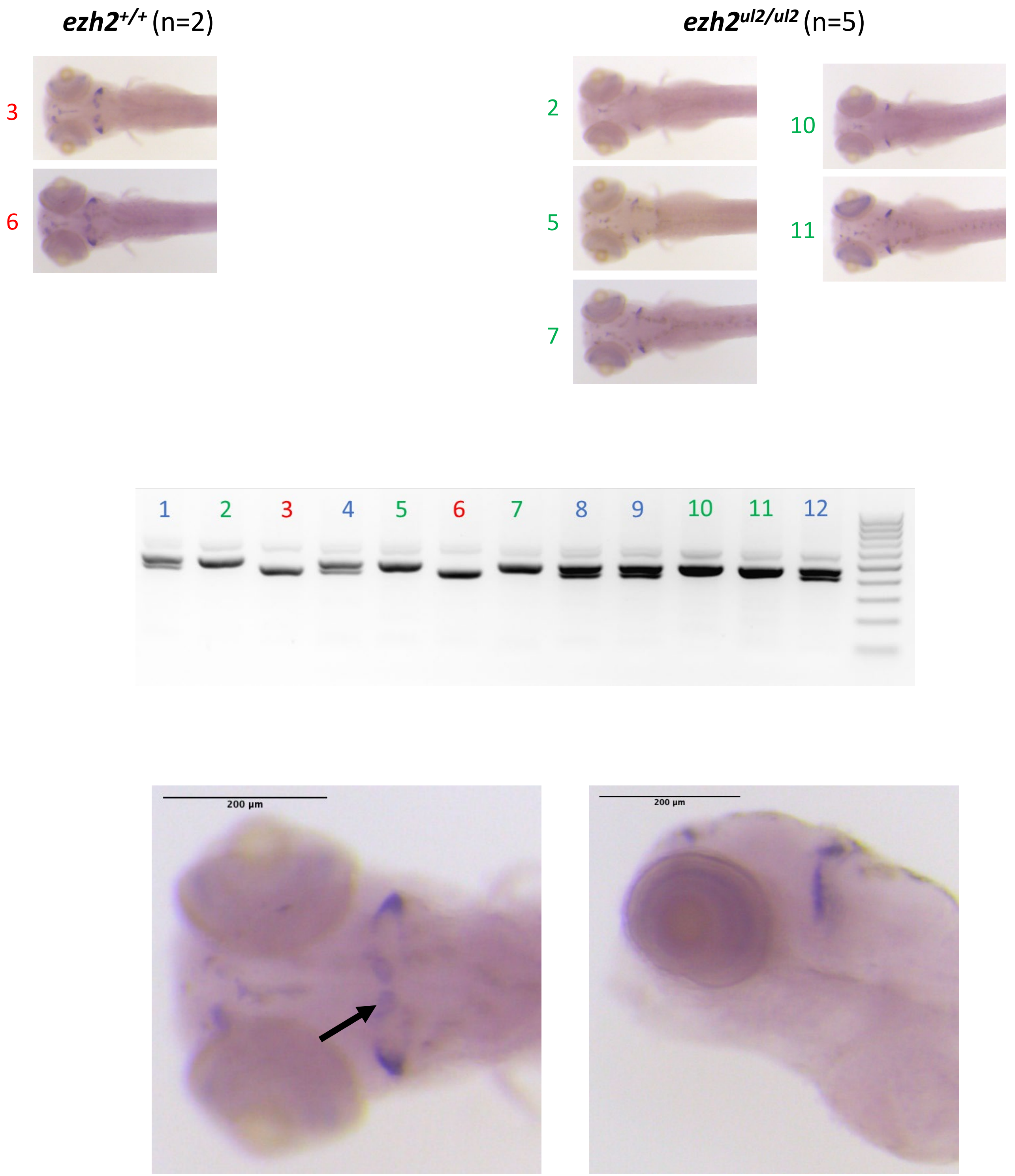

Supplementary Figure S18: Whole-mount RNA *in situ* hybridization for *vglut1* at 5 dpf

pvalb7 at 3 dpf

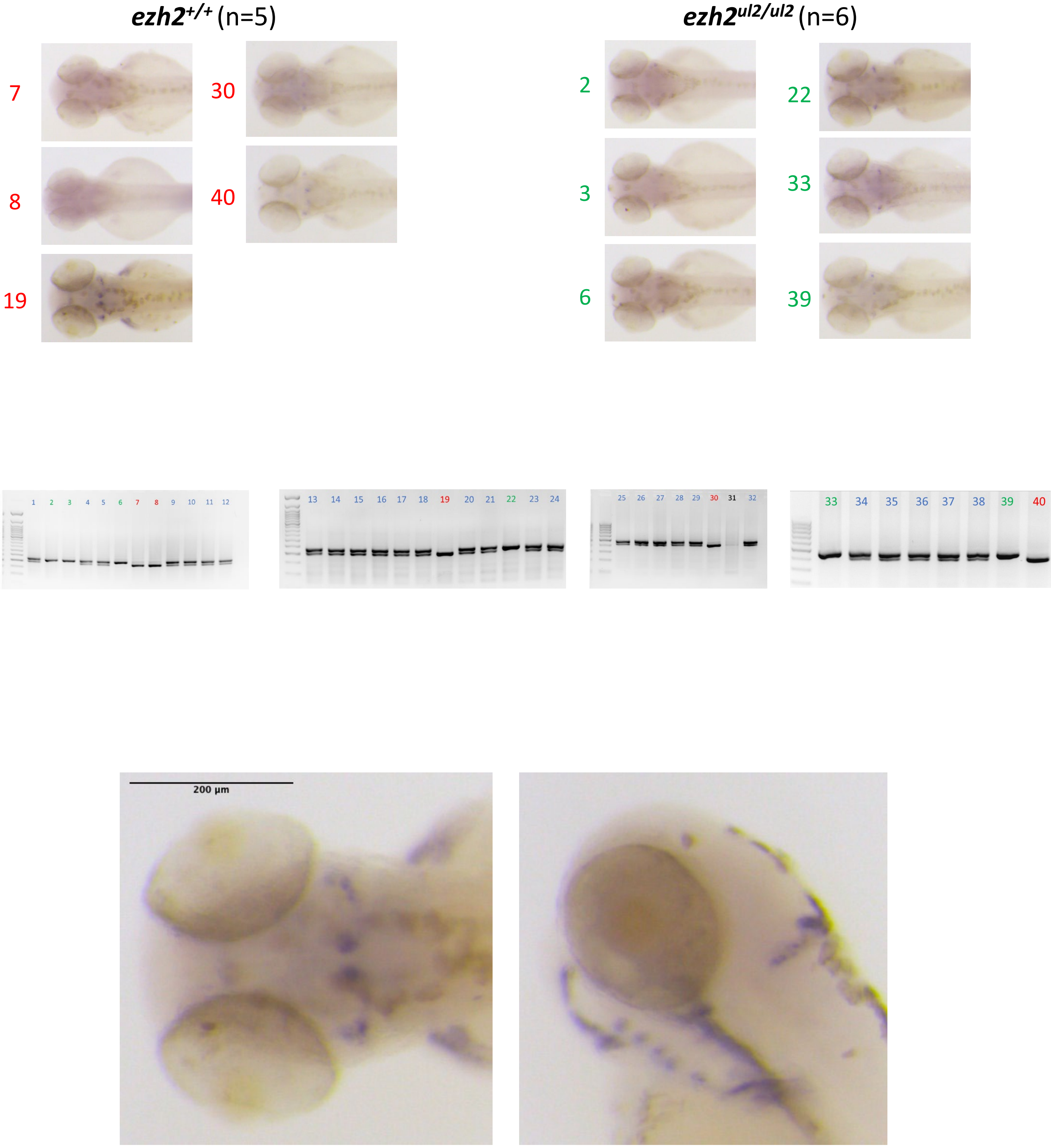

Supplementary Figure S19: Whole-mount RNA *in situ* hybridization for *pvalb7* at 3 dpf

pvalb7 at 5 dpf

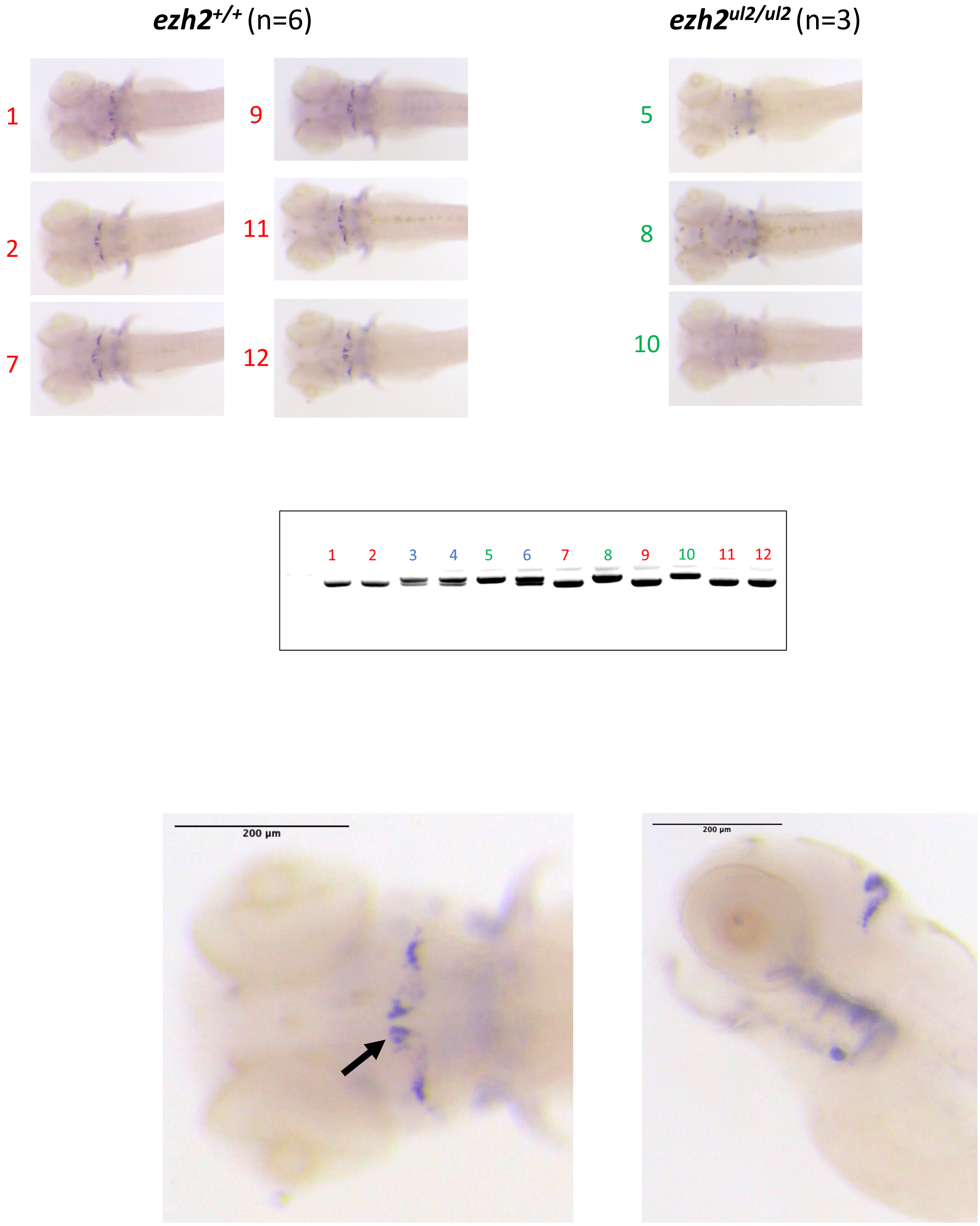

Supplementary Figure S20: Whole-mount RNA *in situ* hybridization for *pvalb7* at 5 dpf

gad1b at 5 dpf

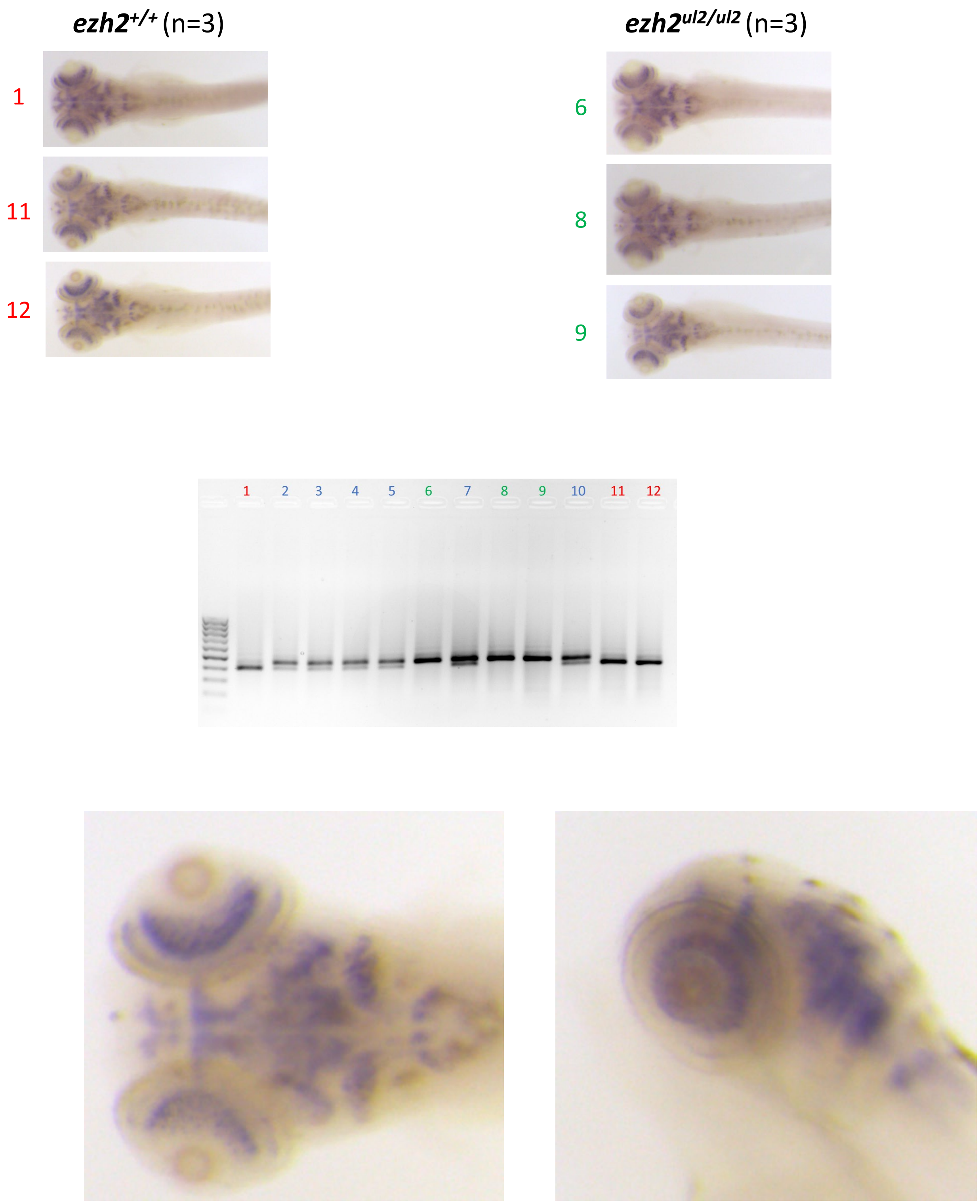

Supplementary Figure S21: Whole-mount RNA *in situ* hybridization for *gad1b* at 5 dpf

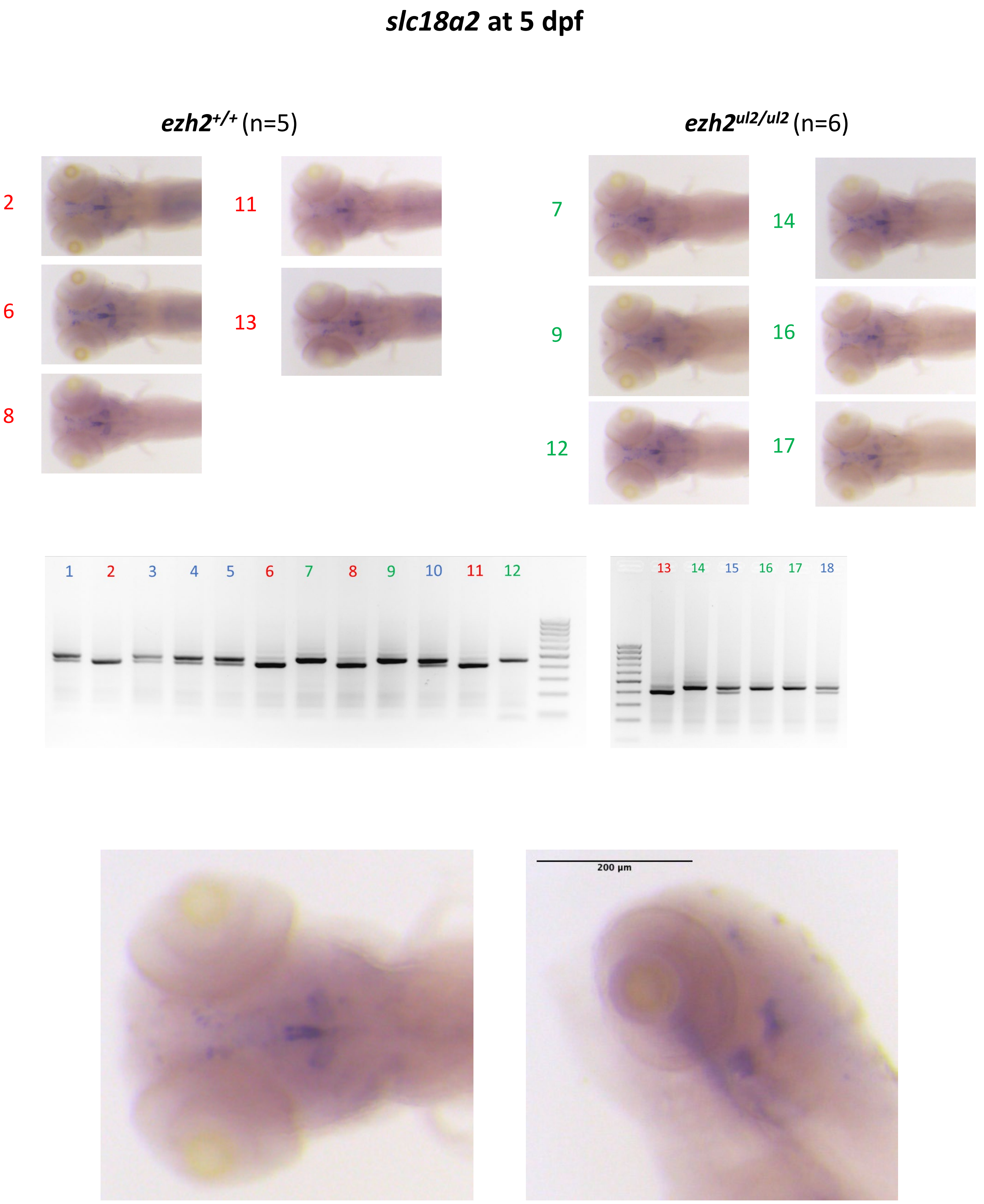

Supplementary Figure S22: Whole-mount RNA *in situ* hybridization for *slc18a2* at 5 dpf

tph2 at 5 dpf

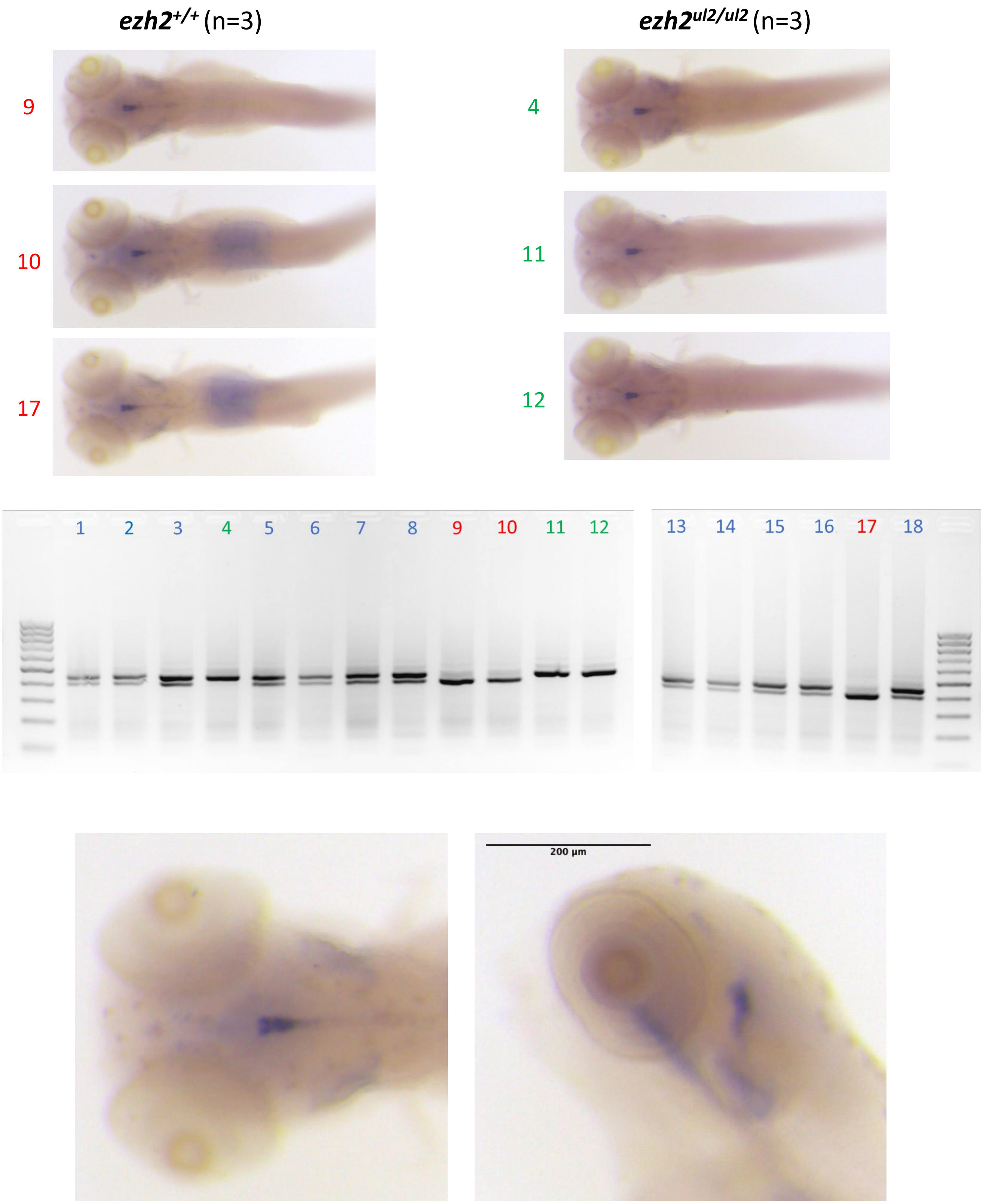

Supplementary Figure S23: Whole-mount RNA *in situ* hybridization for *tph2* at 5 dpf

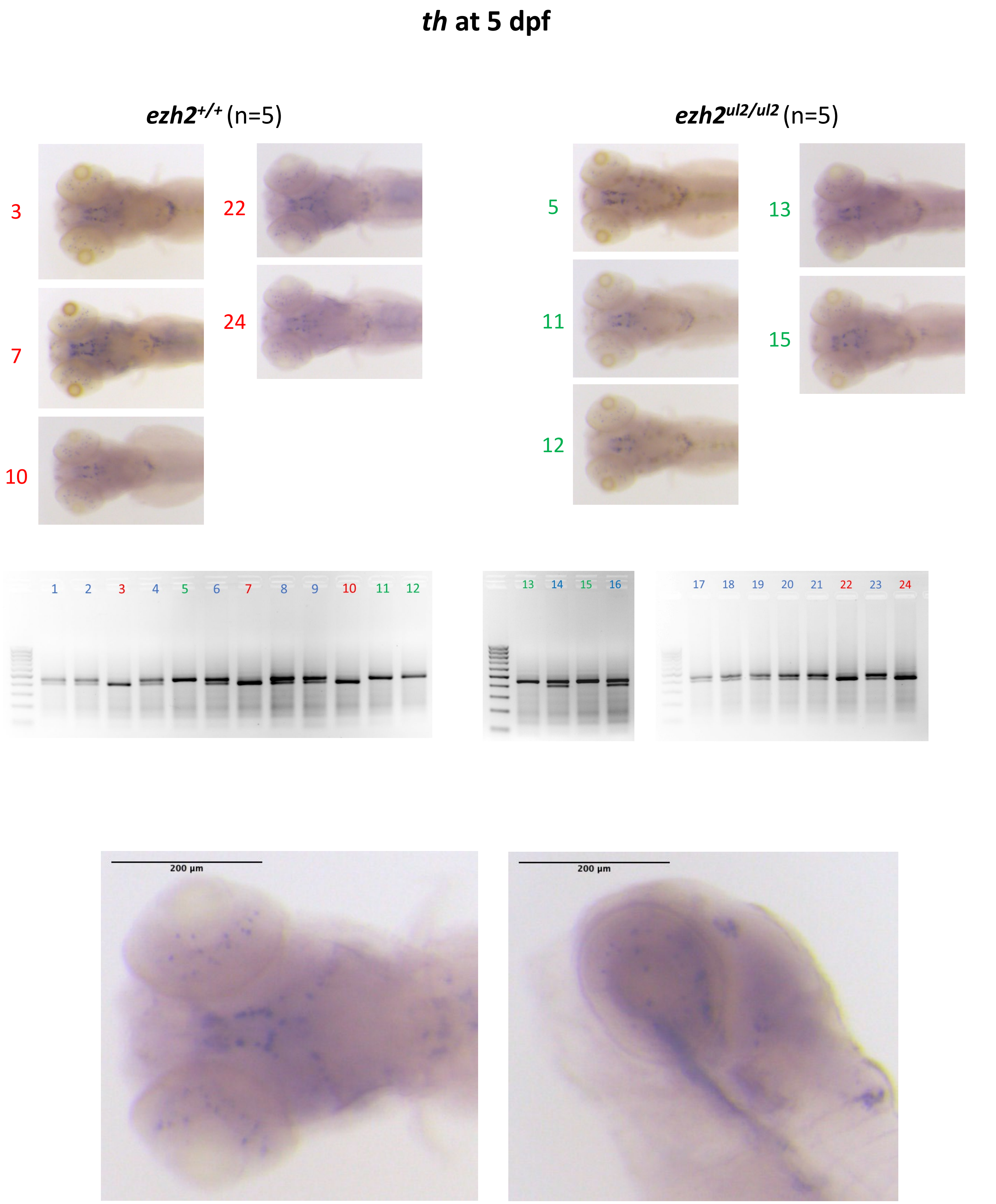

Supplementary Figure S24: Whole-mount RNA *in situ* hybridization for *th* at 5 dpf

ptf1a at 5 dpf

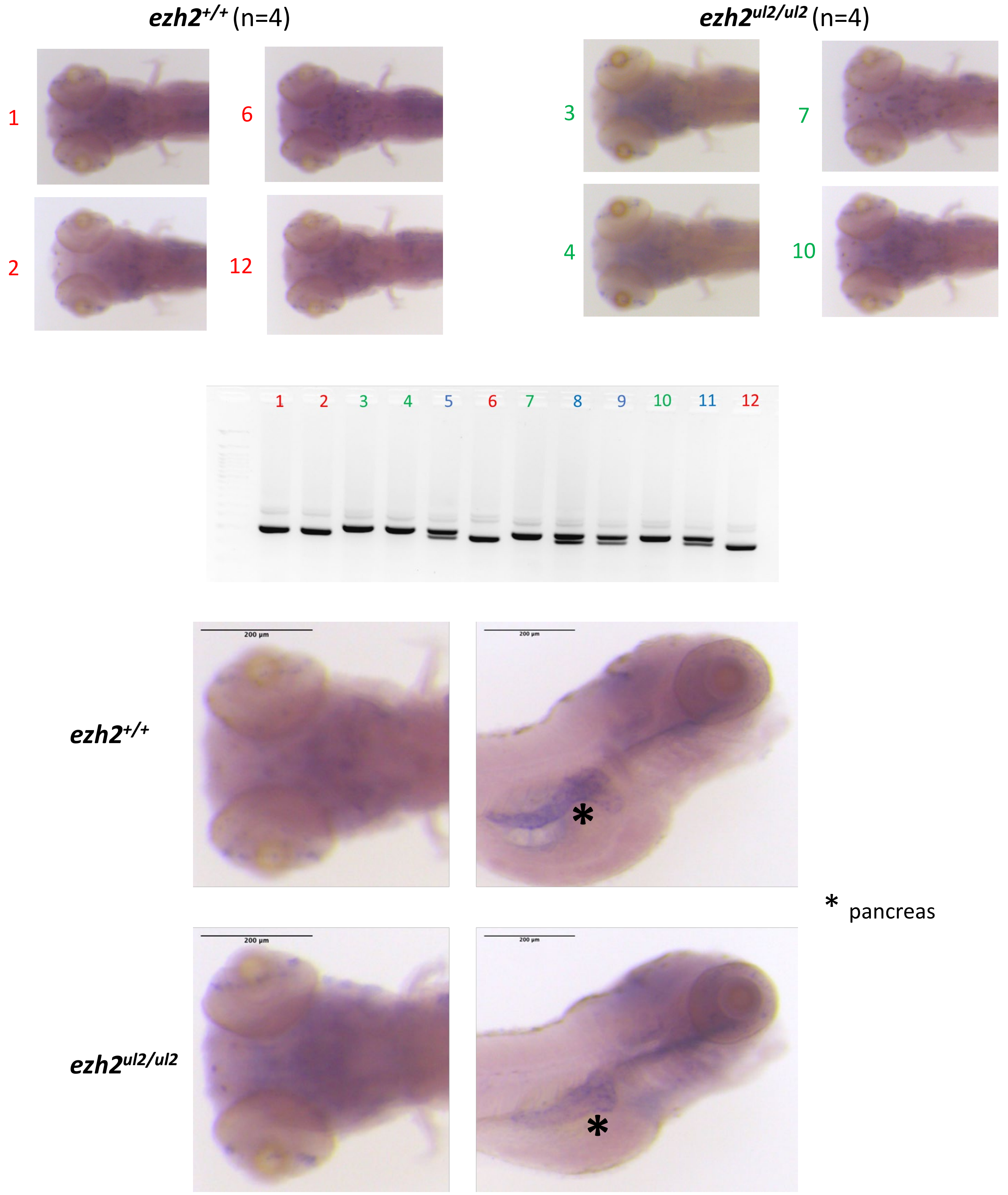

Supplementary Figure S25: Whole-mount RNA *in situ* hybridization for *ptf1a* at 5 dpf

pax2a at 5 dpf

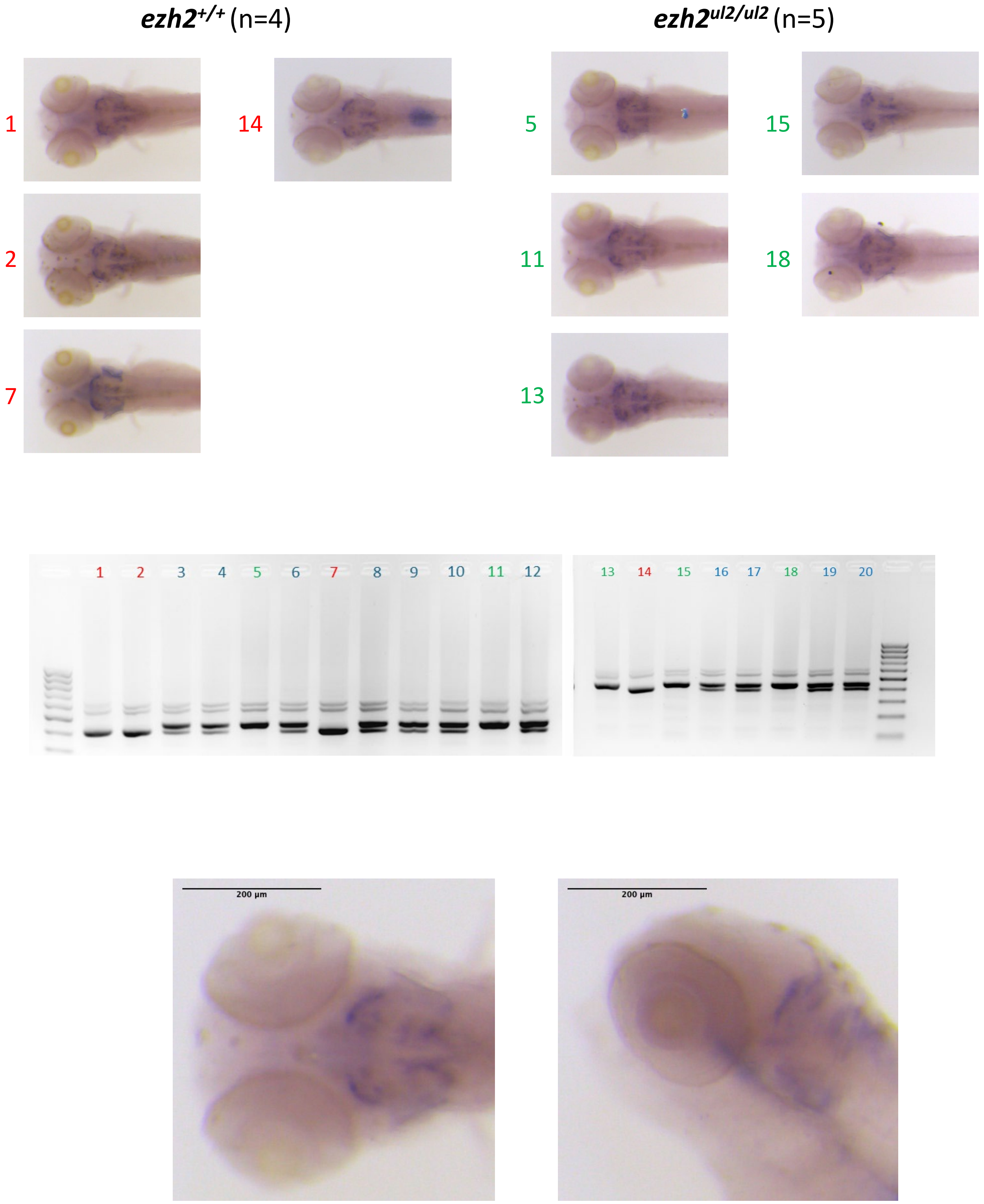

Supplementary Figure S26: Whole-mount RNA *in situ* hybridization for *pax2a* at 5 dpf

EXPERIMENT N° 1

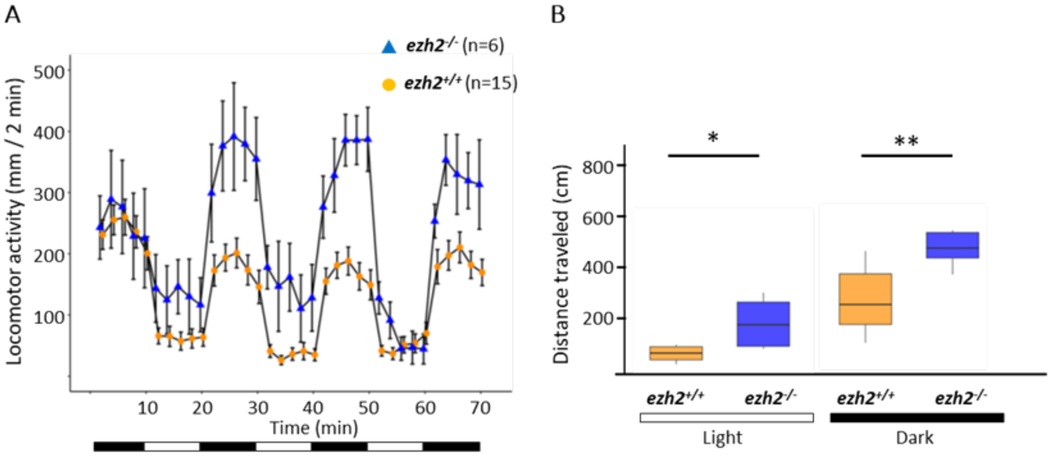

EXPERIMENT N° 2

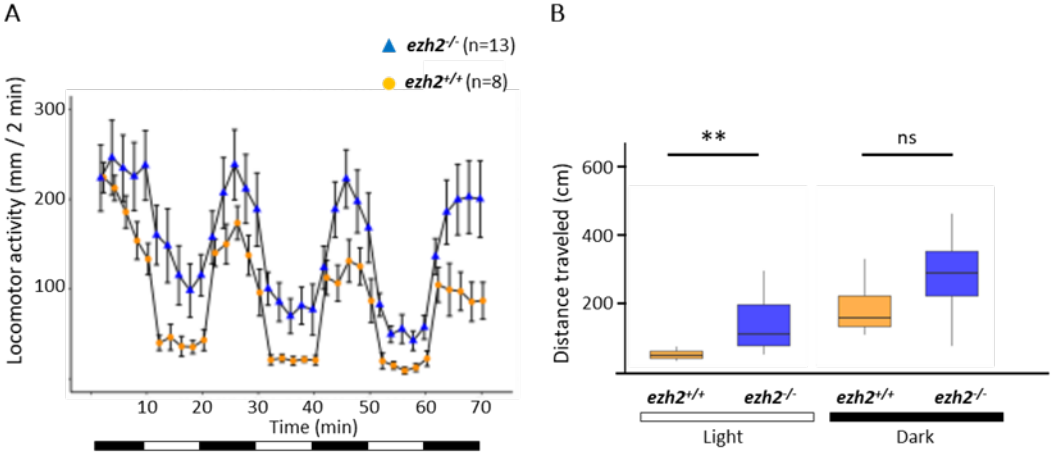

EXPERIMENT N° 3

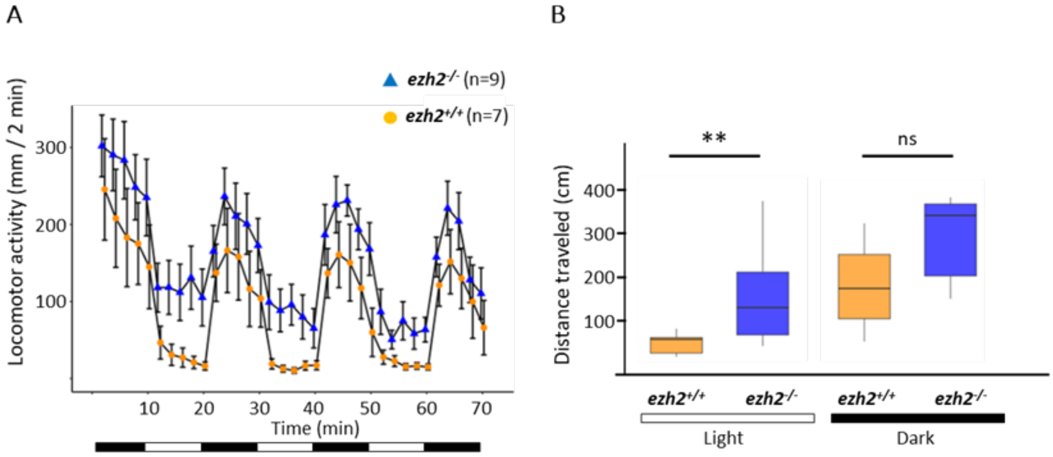

Supplementary Figure S27: Locomotor activity at 5 dpf

EXPERIMENT N° 4

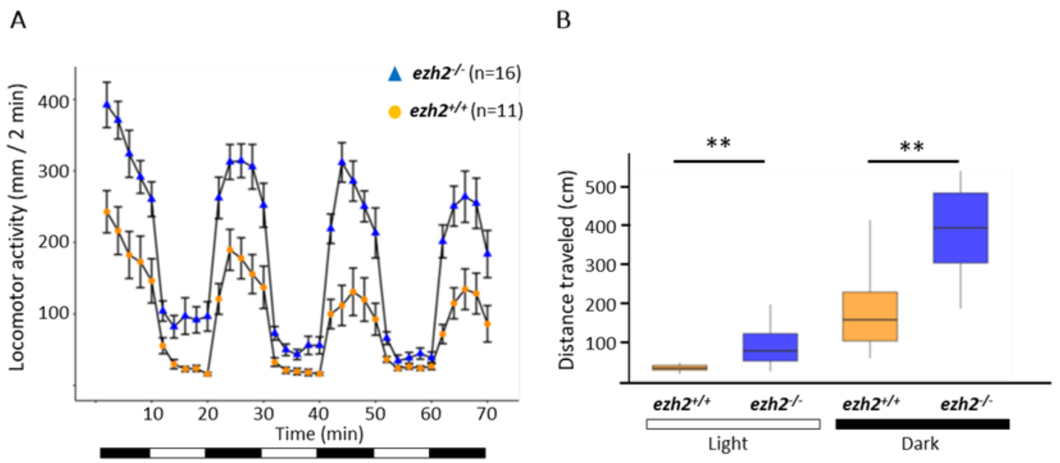

EXPERIMENT N° 5

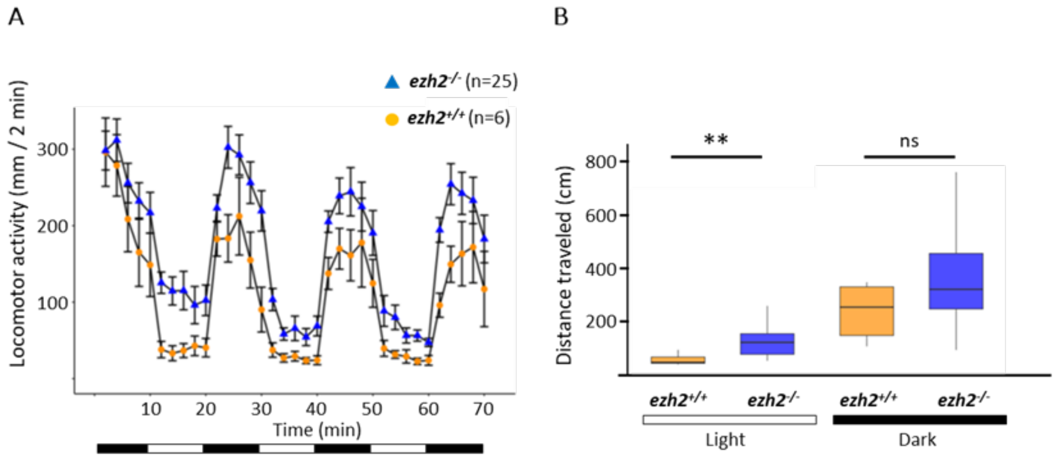

Supplementary Figure S27: Locomotor activity at 5 dpf (Continued)

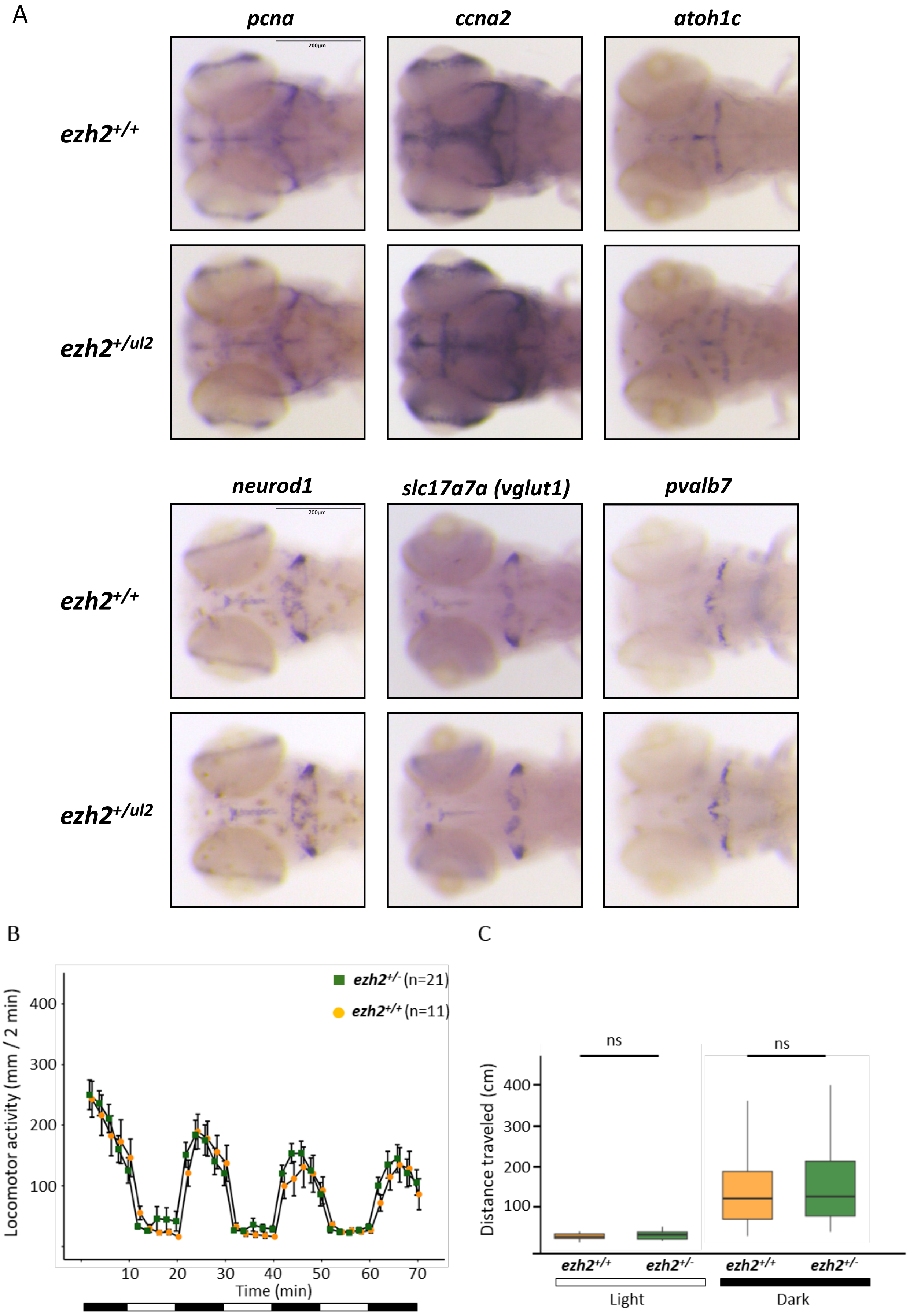

Supplementary Figure S28: Phenotype of *ezh2*$^{+/-}$ heterozygotes
